# Applications of Lysozyme, an Innate Immune Defense Factor, as an Alternative Antibiotic

**DOI:** 10.3390/antibiotics10121534

**Published:** 2021-12-14

**Authors:** Patrizia Ferraboschi, Samuele Ciceri, Paride Grisenti

**Affiliations:** 1 Department of Medical Biotechnology and Translational Medicine, University of Milan, Via C. Saldini 50, 20133 Milano, Italy; patrizia.ferraboschi@unimi.it; 2 Department of Pharmaceutical Sciences, University of Milan, Via L. Mangiagalli 25, 20133 Milano, Italy; samuele.ciceri@unimi.it; 3 Bioseutica BV, Corso Elvezia 4, 6900 Lugano, Switzerland

**Keywords:** innate immunity, peptidoglycan, Gram-positive, Gram-negative, antimicrobial, muramidase, lysozyme

## Abstract

Lysozyme is a ~14 kDa protein present in many mucosal secretions (tears, saliva, and mucus) and tissues of animals and plants, and plays an important role in the innate immunity, providing protection against bacteria, viruses, and fungi. Three main different types of lysozymes are known: the c-type (chicken or conventional type), the g-type (goose type), and the i-type (invertebrate type). It has long been the subject of several applications due to its antimicrobial properties. The problem of antibiotic resistance has stimulated the search for new molecules or new applications of known compounds. The use of lysozyme as an alternative antibiotic is the subject of this review, which covers the results published over the past two decades. This review is focused on the applications of lysozyme in medicine, (the treatment of infectious diseases, wound healing, and anti-biofilm), veterinary, feed, food preservation, and crop protection. It is available from a wide range of sources, in addition to the well-known chicken egg white, and its synergism with other compounds, endowed with antimicrobial activity, are also summarized. An overview of the modified lysozyme applications is provided in the form of tables.

## 1. Introduction

Lysozyme (or muramidase or N-acetylmuramic acid hydrolase E.C. 3.2.1.17) is a protein that exerts its enzymatic activity through the hydrolysis of the β-1,4-glycosidic bonds between N-acetylmuramic acid (NAM) and N-acetylglucosamide (NAG) in the polysaccharide backbone of the peptidoglycans of the Gram-positive bacterial cell wall.

Peptidoglycan is composed of polysaccharide chains cross-linked by short peptides. The polysaccharide chains contain alternate units of NAM and NAG.

**Figure 1:**
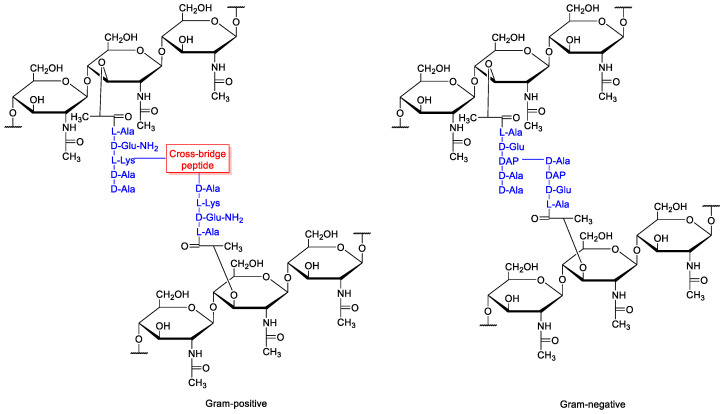
Peptidoglycan composition.

The peptides are bound to the lactate moiety of NAM and usually consist of L-alanine, D-isoglutamic acid (D-isoglutamine in many Gram-positive bacteria), L-lysine or meso-diamino pimelic acid (in Gram-positive and Gram-negative bacteria, respectively), and two D-alanine residues. The side chain peptides of different polysaccharide chains are linked together with a direct link between *meso*-pimelic acid and the first D-alanine (Gram-negative), or through a cross-bridge (Gram-positive), a peptide that is characteristic of each species (Figure 1) [1].

**Figure 2:**
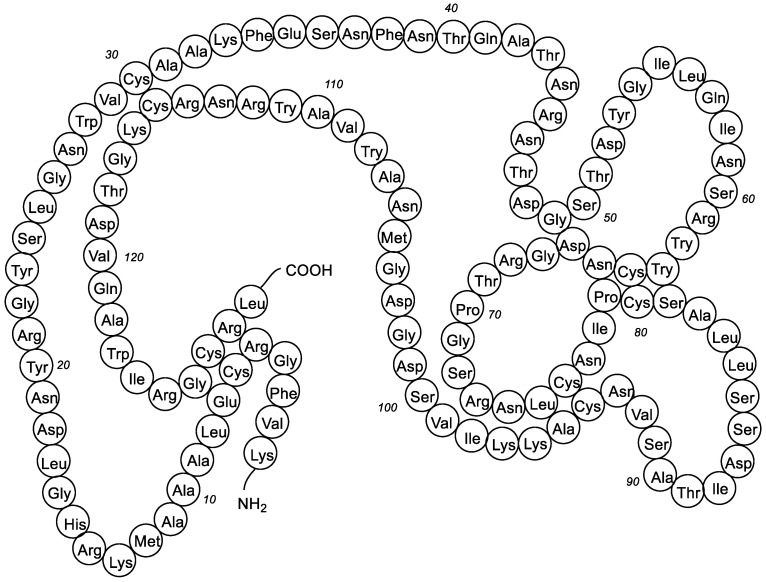
Structure of lysozyme (T. Wu et al. *Food Chemistry* **2019**, *274*, 698–709).

Due to its capacity to disrupt the bacterial cell wall, lysozyme has been considered as an endogenous antibiotic, innately essential in the defense against microbes since its discovery by A. Fleming in 1921 [2]. Lysozyme is a small, monomeric protein stabilized by four disulfide linkages among the eight cysteine residues of its chain (Figure 2).

Lysozymes are present not only in human organs, tissues, and secretion, but also in the organs and secretions of various vertebrates, invertebrates, bacteria, and plants. They are classified into three main families: chicken type (c-type), goose type (g-type) and invertebrate type (i-type) [3]; also known are the phage type, bacterial type, and plant type lysozymes [4,5,6]. The chicken (cLys) and human (hLys) lysozymes are c-type Lys. The cLys is composed of 129 amino acid residues (14.3 kDa), whereas hLys is composed of 130 amino acid residues (14.7 kDa). There is a 59% identity between the sequence of human and chicken lysozymes but the antibacterial activity of hLys is three-fold greater than the antibacterial activity of cLys.

The antibacterial action of lysozyme is particularly efficient against Gram-positive bacteria because of its ability to hydrolyze the β-1,4-glycosidic bond present in the polysaccharide layer of these bacteria cell walls. The effect against the Gram-negative bacteria is significantly weaker due to the presence of a protective lipopolysaccharides layer on the outer membrane.

Many methods, physical and chemical, have been suggested and successfully developed, and aimed at enhancing the susceptibility of Gram-negative bacteria to lysozyme.

Taking into account the well-known antibacterial, antiviral, antifungal, anti-inflammatory, anticancer, and immunomodulatory activity [7], lysozyme has great potential, mainly in clinical, feed, and food applications, for treating pathogens of a different nature, and numerous examples have been reported in literature.

The continuing increase in bacteria resistance to antibiotics prompts the identification of new molecules or new applications of known compounds, such as the case of lysozyme. The chance of the emergence of resistance to peptidoglycan-degrading enzymes, including lysozyme, are described in an interesting 2020 review [1]. The authors explain that Gram-positive bacteria predominantly achieve resistance through peptidoglycan modifications; on the contrary, many Gram-negative bacteria tend to utilize inhibitor proteins that bind the active center of the peptidoglycan-degrading enzymes, blocking their activity. The development of resistance to these enzymes is defined by the authors as a rare event, at least in vitro, emerging not through de novo mutations, but through the horizontal transfer of resistance determinants. The clinical use of peptidoglycan-degrading enzymes is considered as being at less risk of resistance, due to their nature as recombinant proteins.

In the present review, the focus is the more relevant papers and patents published in the last 20 years. The present paper summarizes the main lysozyme source, its application as an alternative antibiotic, its synergism with other compounds, and the physical and chemical modifications aimed to improve its activity.

## 2. Sources

Lysozyme is a ubiquitous enzyme present in all living organisms and viruses with a wide variability in origin, quantity, structural, chemical, and enzymatic properties.

### 2.1. Lysozyme in Eggs

The chicken egg white is the richest source of this enzyme, and it is constituted by about 0.3% of lysozyme. This enzyme accounts for 3.4–5.8% of the total egg white proteins [8,9].

The wide availability makes the chicken egg white the main commercial source for this protein. Many efforts are aimed at obtaining lysozyme in a suitably pure form. In 2018, a review was published about the methods of the purification of lysozyme from egg whites [10]. The purification process of ultrafiltration, according to the authors of a 2009 article [11], extends the spectrum of its activity through the formation of lysozyme polymers. Indeed, it is known that the dimerization produces an enzyme that is active against both the Gram-positive and the Gram-negative bacteria (see the physical modifications Section).

Lysozyme was also found in the hen egg shell membrane, and a 2005 study [12] examined the effect of layer breed, bird age, membrane stabilization treatment, and storage time on the enzymatic and biological activity of lysozyme in egg shell membranes. In a later publication [13], it was observed that there was a lower antibacterial activity of purified lysozyme from egg shell membranes than that of purified lysozyme from egg whites.

Lysozyme from the egg white of quail was purified and characterized in 2014 [14], whereas the polymorphism of the egg white lysozyme from Japanese quail was investigated in 2012, and the authors demonstrated that two phenotypes of lysozyme were associated with significant differences in the antibacterial activity of the enzyme [15].

The g-type lysozyme can be isolated from goose egg whites [16]. It has a three-dimensional structure like the c-type and T4 phage lysozymes.

### 2.2. Lysozyme in Milk

Even though the egg white is the richest source of commercial lysozymes, milk from mammals also contains lysozyme molecules endowed with equally interesting properties. Lysozyme is present in mammals’ milk, either as a free soluble protein or within leucocytes and lysosomes. The lysozymes from milk belong to the c-type family with a wide variability from one species to another in terms of structure, physicochemical properties, and concentrations. Within the same species, the variation depends on many factors (breed, stage of lactation, parturition nutrition, and season of the year), as reported in a 2008 review [17]. A group of the sampled milks contains high levels of lysozyme (200–1330 mg/L) and another group has levels that are 3000 to 6000 times lower. Human, equine, and canine milks belong to the first group while bovine, ovine, and caprine milks represent the second group. In the same 2008 review [17], a table summarized the concentration of lysozyme in different mammals. According to the authors, the low level of lysozyme in milks of some mammals explains the conflicting literature data about the content, the presence, or the absence of lysozyme in the milk of some species (bovine, camel, and porcine).

Jenny milk is characterized by its high lysozyme content and has been used as an antimicrobial additive in dairy products; it is an alternative to the hen egg white lysozyme which can cause allergic reactions [18].

The buffalo milk lysozyme was purified and characterized in 2002 [19], showing a specific activity that was ten-fold that of the bovine milk lysozyme. The sequence of 23 amino acid residues of the N-terminal end was identified, and it showed a 56.5% homology with the bovine milk lysozyme and 30.4% with the equine milk lysozyme.

The N-terminal domain of the human milk lysozyme was treated with pepsin, and the N-terminal helix examined for its antimicrobial activity exhibited a potent bactericidal action to Gram-positive, Gram-negative bacteria, and the fungus *Candida albicans*, showing a potential use for the treatment of infectious diseases [20].

### 2.3. Saliva, Tears, Various Organs, and Tissues of Mammals

Lysozyme was discovered from a casual observation by A. Fleming [2,21,22,23] in 1921, when a few drops of his nasal discharge that contaminated an inoculated culture medium blocked the bacterial growth as observed a few days later. The presence of lysozyme in many forms of human secretion (such as saliva and tears) and tissue and organ secretion (such as placenta, sperm, leukocytes, blood) has been well known since then.

Numerous defense proteins are present in saliva and involved in innate and acquired immunity [24]. The same role is carried out by lysozyme in the lacrimal fluid, and its presence in the retinal pigment epithelial cells has been recently demonstrated [25]: the lysozyme expression is modulated, in this case, by pathogenic challenges.

Interestingly, in 2013 [26], authors from Saudi Arabia demonstrated that lysozyme purified from dromedary tears showed significant bactericidal activity against *Listeria monocytogenes* and *Staphylococcus epidermidis*, whereas the one purified from human tears was devoid of activity against these two strains.

Research about the c-type lysozyme genes in mice allowed the authors to hypothesize the role in mitochondrial functions of spermatozoon and its contribution to the innate immunity of the male genital tract [27].

Ruminant animals have been considered as lysozyme deficient. The expression of lysozyme in the tears, milk, and blood of cows is low. However, in the stomach, the antibacterial lysozymes were recruited as digestive enzymes useful to exploit plant material as a food resource [28]. In a work published in 2010 [29], the yak stomach lysozyme was compared with the cow stomach lysozyme. The result indicated that the yak stomach lysozyme was more closely related to the cow milk lysozyme than to the cow stomach lysozyme. The authors explained this result by concluding that there is a more recent common ancestor of at least one of the stomach lysozymes with milk lysozyme than with other stomach lysozymes. The interest in ruminant animals is also confirmed in a study [30] of a new c-type lysozyme from Lezhi black goat rumen (147 amino acid residues) sharing 70.27% of its identity with the capra hircus blood lysozyme that, likely, functions in host immunity and digestive systems.

### 2.4. Aquatic Organisms

#### 2.4.1. Fish

Lysozyme is present in the mucus, lymphoid tissue, and serum of most fish species but not in cod and wolffish. It has been detected in the oocytes, fertilized eggs, and larval stages of fish species, including coho salmon, sea bass, and tilapia [31].

In aquatic environments, fish are in constant interaction with pathogenic and non-pathogenic microorganisms and, therefore, have developed mechanisms of defense aimed at their survival. The skin layer contains innate and adaptive immune factors that protect against infections. In the skin mucus innate immune factors are higher than in the serum. A study of these factors, comprising of lysozyme, was realized in the skin mucus of five marine teleost fish [32] and three freshwater fish [33], highlighting the variations in the considered fish in order to furnish important information for the aquaculture industry.

Several studies, aimed at identifying amino acid sequences, structure, and antimicrobial activity, focus on the fish lysozyme. The lysozyme from the rock bream (*Oplegnathus fasciatus*) was characterized in 2011 and classified as a g-type lysozyme [34]. In the same year, the lysozymes from kelp grouper (*Epinephelus bruneus*) [35] and from turbot (*Scophthalmus maximus*) [36] were identified and classified as c-type and g-type lysozymes, respectively. Two years later, a c-type lysozyme was isolated and characterized from the leukocytes of a nurse shark [37] and two lysozymes genes, and their recombinant proteins from Asian seabass (*Lates calcarifer*) were analyzed. In this case, either c-type and a g-type lysozymes were identified. The first was most abundant in the liver and the second was predominantly expressed in the intestine and weakly expressed in the muscle [38]. A g-type lysozyme was identified in 2016 [39] in seahorses (*Hippocampus abdominalis*), with the highest expression in the kidney and the least expression in the liver. 

#### 2.4.2. Marine Invertebrates

a. Mollusks

The lysozyme from the viscera of scallops (*Patinopecten yessoensis*) was purified and characterized in 2008 [40]. With the same aim of identifying new, more active enzymes, the lysozymes of other bivalve mollusks, including *Unio pictorum* [41] (four-fold more active than the egg lysozyme in the inhibition of *E. coli*); Asian hard clams (*Meretrix meretrix*) [42]; freshwater mussels (*Cristaria plicata*) [43]; and Manila clams (*Ruditapes philippinarum*) [44], were isolated and identified.

From the mollusk abalone (*Haliotis discus* hannai Ino), a chicken-type lysozyme was obtained and characterized (147 amino acid residues, 15.64 kDa molecular mass, and pI 4.87). This lysozyme showed bacteriolytic activity against Gram-positive and Gram-negative bacteria [45].

b. Crustaceans

The lysozymes from white shrimp (*Panaeus vannamei*) [46,47], black shrimp (*Panaeus monodon*) [48], blue shrimp (*Litopenaeus stylirostris*) [49], and penaeid shrimp (*Marsupenaeus japonicus*) [50] were identified, characterized, and their antibacterial properties analyzed. In some cases, the c-type lysozyme was present while in others the i-type was identified.

The characterization of lysozyme from banana prawn (*Fenneropenaeus merguiensis*) [51] showed a 37–93% similarity with mouse, human, chicken, and tiger prawn counterparts (15 kDa), and a strong inhibition against shrimp pathogens.

c. Echinodermata

The lysozyme of sea cucumbers (*Stichopus japonicus*) was identified in 2009 [52] as an i-type, by cDNA isolation.

d. Anellida

The medicinal leech lives in muddy freshwater pools. In its secretions, from the salivary glands, a multifunctional i-type enzyme, the destabilase-lysozyme, is present. This enzyme is endowed with isopeptidase, muramidase, and antibacterial activity. It attracts interest because it also shows thrombolytic activity through the lysis of the bonds ε-(γ-Glu)-Lys present in fibrin. For these reasons, its recombinant isoforms [53] and antifungal activity [54] have been studied.

### 2.5. Insects

The first antibacterial factor purified from insect hemolymph was lysozyme. The insect hemolymph lysozymes have molecular weights and properties similar to those of the hen egg white lysozyme, but a higher enzymatic activity.

*Cameraria ohridella* is the most dangerous pest to the horse chestnut. In 2005, [55] the lysozyme-type activity of the pupae of this insect was identified against *Micrococcus luteus* and *Bacillus megaterium*. Additionally, the lysozyme c-1 of *Anopheles gambiae*, in the course of its characterization, inhibited the growth of *M. luteus* but not of *E. coli* [56]. The possibility of cloning and overexpressing the lysozyme of *Spodoptera litura* in *E. coli* offers a method for the production of the biologically active c-type lysozyme as a natural antibiotic [57,58]. The same authors applied a similar process to the overexpression of lysozyme from *Agrius convolvuli* obtaining a peptide active against *B. megaterium* and *M. luteus* [59]. 

The larvae of *Galleria melonella*, the honeycomb moth, parasitize the honeybees, and the economic loss caused by this species prompted numerous studies. The c-type lysozyme of *G. melonella* is endowed with antifungal activity against *Candida albicans*, and the mechanism of this action was investigated by a Polish group in 2016 [60].

The awareness of the presence of lysozyme in the hemolymph of honeybees has existed since 1968 [61], and the antimicrobial properties of honey alone [62] or in combination with milk [63] have been reviewed.

A new type of lysozyme from the Chinese oak silk moth (*Antheraea pernyi*) was investigated and the encouraging results obtained about the strong effectiveness against Gram-negative strains, according to the authors, laid the foundation for future improvement by protein engineering [64].

Moreover, the c-type lysozyme from the Asian corn borer (*Ostrinia furnacalis*) [65] showed to be active against Gram-positive and Gram-negative bacteria.

Recently, a c-type lysozyme from *Coridius chinensis*, a medicinal insect resource in China, was identified and analyzed [66].

### 2.6. Plants

A novel plant lysozyme was isolated in 2005 [67] from the mung bean (*Phaseolus mungo*), with a molecular weight of 14.4 kDa, and exhibited antifungal activity toward *Fusarium solani*, *Pythium aphanidermatum*, *Sclerotium rolfsii*, and *Botrytis cinerea*, and antibacterial action against *Staphylococcus aureus*. A similar antifungal and antibacterial activity was found in the case of lysozyme isolated from Canadian cranberry beans (*Phaseolus vulgaris*) [68].

The seed oil from *Carthamus tinctorius* safflower [69] and the milky juice of papaya fruits [70] are rich sources of proteolytic enzymes, including lysozyme. For this reason, they are applied in the treatment of various skin injuries.

The lysozyme isolated from *Momordica charantia L.* was found to exhibit antifungal activity toward *Mucor racemosus* and *Rhizoctonia solani*, in addition to the antibacterial action against *E. coli* and *S. aureus* [71].

### 2.7. Microorganisms

The research of new antibacterial drugs able to overcome the antibiotic-resistant bacteria problem, also focused the attention toward bacteria, bacteriophages, and yeast as sources of new lysozymes.

Recently [72], a Chinese patent reported the identification of a bacteriophage lysozyme and its gene, as well as its use for preventing and treating bacterial infections.

*Pichia pastoris* is a methylotrophic yeast which has proven to be an efficient system for the expression of many heterologous proteins. The DNA of an unstable mutant in the hen egg lysozyme was integrated in *P. pastoris*, and the amount of secreted enzyme was 422-fold greater than what was observed with *Saccharomyces cerevisiae* [73]. The same yeast was employed to insert the T4 lysozyme gene, and the obtained protein inhibited the growth of *S. aureus* and *Streptococcus pneumoniae* [74]. In another example, *P. pastoris* was modified with the gene encoding the lysozyme. The produced lysozyme, in the presence of silicic acid, mediated the encapsulation of yeast cells within silica, paving a novel way for the preparation of composites, finalized to biotechnological applications [75].

The lysozyme of *Bacillus licheniformis* from soil was cloned and expressed in *E. coli.* The produced lysozyme was resistant to pepsin and trypsin, to some extent at, 40 °C, and efficiently active in the pH range between 3 and 9 and from 20° to 60 °C, respectively. The promising properties of this preparation [76] as a food or feed additives was also found for the fungal lysozyme isolated from *Trichoderma reesei*, showing an antimicrobial activity improved at the acidic pH (<6.5) [77].

According to the authors [78], another fungal lysozyme, produced by the Chalaropsis species, could be utilized in a variety of settings where bacterial infections proliferate, such as hospital settings (*S. aureus*) or in veterinary applications (mastitis from *S. aureus* in cows), and as a means of combating bioterror agents (such as *Staphylococcal Enterotoxin B* and *Clostridium botulinum*).

Chitinases are the enzymes which hydrolyze chitin, the β-1,4-linear polymer of N-acetylglucosamine, one of the most abundant natural polysaccharides. A bifunctional chitinase/lysozyme from *Bacillus pumilus*, capable of degrading the chitin component of fungal cell walls and the peptidoglycan component of cell walls of many kinds of bacteria (*Xanthomonas translucens*, *Xanthomonas axonopodis*, *Bacillus licheniformis*, *E. coli* C600, *E. coli* TOP10, *Pseudomonas aeruginosa*, and *Pseudomonas putida*), was cloned and expressed in the *E. coli* strain M15 [79]. The chitinases from plants and animals are frequently endowed with lysozyme activity, whereas the bifunctionality of microbial chitinases is rare.

### 2.8. Recombinant Human Lysozyme (rhLys)

The great antimicrobial activity of lysozyme makes it interesting in medicine, cosmetics, and the food industry. The chicken egg white lysozyme is commonly used for these purposes but individuals sensitive to chicken eggs have also demonstrated an allergic reaction to the lysozyme isolated from egg whites. Many investigations have been carried out to produce the human lysozyme, considering its limited source, in bacteria, yeast, plants, and other organisms. In a 2006 patent [80], the preparation and the purification of rhLys with a transgenic organism, *E. coli*, was described. The obtained protein exhibited enzymatically active bacteriolytic properties, showing that *E. coli* can produce a functionally active human lysozyme. A bioreactor, with plastic composite support, was used to optimize the growth parameters of *Kluyveromyces lactis* K7, a genetically modified organism that expresses the human lysozyme [81].

A novel human c-type lysozyme was produced in recombinant *Pichia pastoris* using a fed-batch strategy; the obtained lysozyme showed a specific activity toward *Micrococcus lysodeikticus* of 7069 U/mg (*Micrococcus lysodeikticus* is a Gram-positive organism, isolated by A. Fleming when he discovered lysozyme. It is the standard microorganism utilized for the evaluation of muramidase activity. The current name for *M. lysodeikticus* is *M. luteus [82]*; in this review, we have used the former name or the new one, depending on the choice by the authors of the cited articles), suggesting a possible industrial application [83]. The expression of rhLys in *Pichia pastoris*, fused with the peptide tachyplesin I, was reviewed in 2013 [84]. 

Lysozyme is highly expressed in human milk but is found only in trace amounts in cow’s milk. In a work conducted in 2011, 17 healthy cloned cattle expressing the recombinant human lysozyme were produced. The transgenic cattle milk offered similar nutritional benefits as human milk, and the described techniques are appliable for the production of the active human lysozyme on a large scale [85]. More recently, recombinant human lactoferrin and lysozyme were produced and characterized in a bi-transgenic cow. The enzymatic activity of the lysozyme in the transgenic milk was comparable to that of human milk, which is 6 and 10 times higher than that of the bovine lysozyme present in milk [86].

A bovine mammary gland expression vector, expressing the human lysozyme gene, was constructed and tested on lactation rabbits [87] and in mouse mammary epithelial cells [88]. Transgenic mice were also developed for the expression of large amounts (18.5–35 g/L) of rhLys in milk [89], representing a model system for the cost-effective production of hLys. I previous studies, significantly lower amounts of lysozyme (1.20–1.76 g/L) were obtained using similar approaches [90,91,92].

Transgenic swine expressing rhLys were generated by a somatic cell transfer, with the aim to feed piglets with the human lysozyme to avoid pathogenic infections and, hence, a negative impact on neonatal survival. One of the 3 cloned female pigs expressed rhLys at 0.32 μg/mL in milk, 50-fold higher than the native pig lysozyme [93].

Transgenic dairy goats that expressed the human lysozyme in their milk, at 68% of the level normally found in human milk, were developed, in order to extend the beneficial protective properties of human milk into livestock milk and make it readily available for people of all ages [94,95].

Both the chicken egg lysozyme and human lysozyme belong to the c-type. Although the chicken egg lysozyme is easily obtained from egg whites, hLys displays a 3-fold higher antibacterial activity and is more thermal stable. For these reasons, transgenic chickens suitable for the production of active rhLys, were generated and the analysis of the obtained rhLys showed physicochemical and biological properties similar to commercial hLys. Moreover, the transgene of rhLys was genetically stable across the different generations [96].

Th breast feeding of fresh human milk has traditionally been considered the best means for providing nutrition to infants; indeed, it has been demonstrated that lysozyme and other milk proteins are immune factors that compensate for the undeveloped defense mechanism of the gut of infants [97]. For the situations in which the mother’s milk is not available, synthetic infant milk formulas are used in the place of breast feeding. In this context, the expression of human milk proteins (including lysozyme) in transgenic plants was developed.

A synthetic gene of hLys was introduced into the calli of rice [98]. The obtained rhLys was purified and the amino acid sequence verified, showing the promising potential for using a rice-derived lysozyme as a food supplement for infant formula and baby foods. The transgenic rice expressing lactoferrin and lysozyme was fed to chicks, showing antibiotic-like properties similar to subtherapeutic doses of baritrocin + roxarsone in the protection of the intestinal tract [99].

## 3. Applications of Lysozyme

The large number of investigated sources of different types of lysozymes can be explained by the many applications in medicine, cosmetics, the food industry, and agriculture. The wide spectrum of applications depends not only on its antibacterial activity, but also on the inactivation of certain viruses and fungi.

The antibacterial activity against Gram-positive bacteria has been explained by the lysozyme enzymatic action on the peptidoglycans present in the cell wall. The peptidoglycans present in the inner membrane of Gram-negative bacteria are shielded by a lipidic outer membrane, but the lysozyme shows, even if weakly, to be active. Some authors explained this activity, proposing that the antibacterial mechanism of action is independent of its enzymatic activity, also in the case of Gram-positive bacteria. The role of the lysozyme, according to this hypothesis, is the removal of the cell wall of the bacteria previously killed by antimicrobial polypeptides. Ibrahim et al., in 2001 [100], showed that the catalytically inactive mutant of the hen egg white lysozyme was as bactericidal as the wild -type lysozyme against *S. aureus* and *B. subtilis*. An opposite opinion was suggested in the same year by Masschlck et al. [101], who observed that a high pressure treatment, in the presence of lysozyme, sensitized a series of Gram-negative bacteria; the denaturation of lysozyme, by heat treatment, fully eliminated the bactericidal effect observed under high pressure conditions, while a partially denatured lysozyme maintained its activity. The bactericidal effect, due to the high-pressure treatment, was observed also in the case of two peptides, devoid of enzymatic activity, obtained from lysozyme: the authors ascribed these results to the cationic nature and the increased hydrophobicity of the chains.

The role of cationic peptide chains (the depolarization and permeabilization of membranes) was discussed in an article published in 2004 [102], together with the hypothesis of the indirect bactericidal action of lysozyme: the cationic peptide can behave as antibacterial by activating an autolytic wall muramidase of bacteria (a phenomenon defined as “Trojan horse”), resulting in bacteriolysis. 

The action mechanism of lysozyme was studied also in vivo to verify if its activity depends or not on the muramidase action. From the observed results in transgenic mice deficient in lysozyme or expressing a muramidase-deficient lysozyme transgene, the authors concluded that lysozyme kills bacteria independently of its muramidase activity [103]. 

The cationic nature of the lysozyme chain was hypothesized to be the cause of its fungicidal activity. The ionic interactions between the cationic peptide and the anionic structures in the microbial cell wall can result in the damage to the cell wall, which is disrupted by a subsequent event, such as the exposure to salt and detergent by the effect of osmotic pressure. [104].

In an article published in 1999, the presence of a protein with the N-terminal 15 amino acids sequence identical to the human urinary lysozyme C in preparations of the β-subunit of human chorionic gonadotropin, was reported. The antiviral activity of this protein and of the lysozymes from chicken egg whites, from human milk, and from human neutrophils against HIV-1, was explained by the authors as being due to the degradation of viral polysaccharides [105].

The number of suggested potential mechanisms of action is as wide as the field of lysozyme applications against microorganisms, which differ greatly from one another.

### 3.1. Medical Applications

#### 3.1.1. Skin Diseases

The milky juice of papaya fruits is a source of proteolytic enzymes, including lysozyme, and it is applied in surgery for the treatment of fistulas, cleaning wounds from necrotized tissues, and for skin grafting [70].

High antibacterial effects produced by both bacteriostatic and bactericidal pathways, including lysozyme activity, was demonstrated for the seed oil from *Carthamus tinctorius* (safflower) [69], in the management of skin injuries.

*Staphylococcus aureus* is the most common cause of primary and post-operative skin infections, and it has become increasingly resistant to antibiotics, such as methicillin and vancomycin. The use of lysozyme from egg whites and its dextran conjugate was investigated as an alternative topical ointment for the treatment of the infected skin of mice [106]. The two preparations were tested in vitro against *S. aureus* and *E. coli*. The results showed that both the lysozyme and lysozyme conjugate exhibited antibacterial activity against *S. aureus*, but only the lysozyme conjugate was active against *E. coli*. The activity of the conjugated lysozyme was explained by the authors by the strong surface activity which can enhance the lytic action of the enzyme toward the peptidoglycan layer in the inner membrane. The studies on mice also confirmed the improvement of the antibacterial activity of the lysozyme in wound healing, due to the conjugation with dextran. The activity of the dextran conjugated lysozyme was comparable with tetracycline, suggesting that lysozyme is a natural antimicrobial agent and a suitable replacement for synthetic antibiotic.

**Figure 3:**
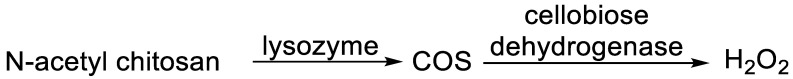
Antimicrobial system involving lysozyme.

In a 2017 publication [107], a smart antimicrobial system, activated in the case of infection, based on elevated lysozyme activity, was presented. A synthesized N-acetyl chitosan was subjected to the lysozyme hydrolysis in artificial wound fluid, presenting N-acetylated chitooligosaccharides (COS). COS, by action of cellobiose dehydrogenase, afforded antimicrobial hydrogen peroxide (1 mM), which is able to inhibit the growth of *E. coli* and *S. aureus* (Figure 3).

A T4 lysozyme fused with a cellulose binding module was prepared and immobilized to a wound dressing gauze. The immobilized protein retained the bacterial activity against Gram-positive and Gram-negative bacteria. The unmodified T4 lysozyme could not bind to the gauze. The immobilized lysozyme can constitute an innovative strategy for producing antimicrobial wound dressing materials [108].

Acticoat, an antibacterial silver nanoparticle-loaded dressing, is a commonplace for the prevention of infection in burns and with open wound patients. The efficacy of this dressing against methicillin-resistant *S. aureus* (MRSA) was evaluated, investigating additives that can improve its activity [109]. The greatest reduction in bacterial survival was observed when Acticoat was soaked with a combination of 10% glycerol, lysozyme (1 mg/mL), and an antimicrobial peptide (bac8c, a truncated and modified bovine neutrophile peptide).

A promising preparation for the development of antibacterial wound dressing was obtained in 2018 [110], starting from hairy steric stabilized nanocrystalline cellulose (SNCC) functionalized with aldehyde groups; by the reaction of these groups, lysozyme or nisin was immobilized on cellulose. Lysozyme and nisin in free and immobilized forms were tested against *B. subtilis* and *S. aureus*. *S. aureus* is the bacterial species more commonly detected in infected wounds and *B. subtilis* is closely related to several animal pathogens, including *B. cereus*, which is associated with wound infections. Immobilized nisin showed to be active against *S. aureus*, whereas free nisin became ineffective against the growth of *S. aureus* after 24 h. Lysozyme was not effective against *S. aureus*, but the immobilized lysozyme was active against *B. subtilis*. The authors of the study suggest that the combination of antimicrobial agents immobilized onto SNCC can offer an effective broad spectrum antibacterial wound dressing.

Recently [111], the effect of wound moisture on wound healing was studied considering the moisture balance of a polyurethane foam dressing. A moisture balanced antibacterial dressing was constructed by loading lysozyme onto a polyurethane foam dressing, by means of dopamine adsorption. The prepared dressing experiment in wound healing in infected mice provided the appropriate wound moisture and at the same time prevented bacterial infections.

The most common skin disorder is the acne vulgaris caused by *Propionobacterium acnes*. The use of lysozyme-shelled microbubbles (MBs) and ultrasound-mediated Lys-MBs cavitation against *P. acnes*, in vitro and in vivo, aimed to reduce the dose and the duration of antibiotic therapy, was investigated [112]. The results of the study showed that the combined Lys-MBs and ultrasound significantly reduced the treatment duration and inhibited *P. acnes*-induced skin diseases.

A different approach to the control of *P. acnes* by lysozyme was proposed in 2018. Bacteriocin AS-48 is a 70-amino acid residue circular peptide produced by different *Enterococcus* species, endowed with bactericidal activity on many Gram-positive and Gram-negative bacteria. The effectiveness against *P. acnes* by AS-48 alone, and in combination with lysozyme, was examined using a range of microscopy and bioassay techniques. The improvements of the action of AS-48 through the combination with lysozyme showed that these two natural compounds are promising candidates against dermatological diseases, such as acne vulgaris [113].

The use of a lysozyme gel formulation in the disinfection of the skin, during pre- and post-surgery, for facial care and the care of hands, feet, and nails, was reported in a 2013 U.S. Patent [114]. The gelled lysozyme was prepared by the addition of water to a suspension of lysozyme in alcohol, without the addition of other gelling substances. The formulation, retaining the enzymatic activity, was successfully used for local applications in the pre- and post-operative therapy of phlebopathic patients.

A polyethylene-based material loaded with an antibiotic is often used as a surgical sealant, but the developed drug resistance prompted the development of a variety of bioactive molecules modified by a PEG-based hydrogel. The antibacterial activity of lysozyme prompted the development of a PEG-lysozyme injectable sealant. A four-arm PEG suitably functionalized at each terminal arm was linked to lysozyme through its amine groups. It was observed that the hydrogel sealed gas or blood leakage in a rabbit trachea, and it could close the transmural left ventricular wall defect. The bacteriostatic activity was demonstrated against *S. aureus* and *E. coli* [115].

#### 3.1.2. Medical Devices

Bone tissue engineering plays a crucial role in regenerating defective or lost tissue. The selection of material is important since it is necessary to consider the biological properties of living cells and the physicochemical properties of the materials. A silica precursor was blended with a SiO_2_-CaO-P_2_O_5_ mesoporous bioactive glass solution. Lysozyme was encapsulated into the scaffold, which exhibited an ability to delay the pH-triggered lysozyme release endowed with antibiotic activity [116].

A silicon rubber film modified with N-vinyl caprolactam was prepared with the aim to reversibly load and release lysozyme for medical devices. The enzymatic activity was assessed against *Micrococcus lysodeikticus* [117]. 

Pelvic organ prolapse is a benign post-partum gynecological disease which seriously affects the quality of life of middle-aged and elderly women. Mesh, mainly polypropylene, is a material used for pelvic floor reconstruction. The postoperative complications prompt to find alternative materials. In a recent article [118], lysozyme and collagen were alternatively deposited on silk fibroin and nylon 6 composite nanofibrous mats through a layer-by-layer self-assembly method. Silk fibroin was chosen for its biological compatibility, biodegradability, and low immunogenicity. The poor mechanical properties of silk fibroin nanofibers were overcome by blending silk fibroin with nylon 6, a synthetic polymer with excellent mechanical strength. These mats showed an over 98% reduction in the viable count of *S. aureus*. The less encouraging results against *E. coli* can be explained because lysozyme is less sensitive to Gram-negative bacteria.

The immobilization of lysozyme onto woven or knitted PET [119], decreased the adhesion of vein catheter-isolated *Staphylococcus epidermis* by 6- to 8-fold and of *S. aureus* by 11- to 12-fold, while the Gram-negative *E. coli* showed a decrease of 3- to 4-folds. Immobilized lysozyme on PET can inhibit the graft-associated infection.

The oral healthcare of aged people is an issue of great interest and importance due to ageing societies. The development of self-cleaning materials is closely related to the adsorption phenomena on both dental material and teeth of antibacterial agents. These agents can be synthetic compounds or naturally secreted substances, such as lysozyme. The quartz crystal microbalance method associated with dissipation monitoring is a technique suitable for sensing the “softness”, or viscoelastic properties, of the adsorbed layer. Through this method, the adsorption of lysozyme on the Au sensor, SiO_2_ sensor, or TiO_2_ sensor, were studied at different pH conditions and salt concentrations [120]. The affinity of lysozyme was in the order of Au > SiO_2_ > TiO_2_ sensors. The pH-dependent charge of oxide surfaces showed to influence the lysozyme adsorption, suggesting that the nature of the adsorption on the oxide surface was electrostatic. 

The osteointegration of dental implants with surrounding tissue and the presence of infection at the implant site are critical factors in the success of dental implants. 

Graphene oxide, a nanosheet with a thickness of several atoms, has a large surface area and it is rich in functional groups. It possesses an appreciable ability to kill Gram-positive and Gram-negative bacteria, due to its behavior that resembles a nano knife being able to penetrate and cut the cell membrane. However, this nonspecific mechanism also affects other cells, leading to a biotoxicity strictly related to the concentration. In 2020 [121], graphene oxide was integrated with lysozyme and tannic acid into a thin coating using a layer-by-layer method. The tannic acid extracted from plants has antibacterial properties and strong osteogenesis, due to its binding to calcium ions. Subsequently, the new coating was characterized by the beneficial effects of the integrated components. The layer-by-layer method allowed us to overcome the concentration-dependent graphene oxide toxicity because a very low amount was required to cover the entire substrate. The efficacy of this coating was demonstrated against the Gram-positive *S. aureus* and the Gram-negative *E. coli*. 

A lysozyme coating was proposed again in 2020 [122], with the aim to improve the corrosion resistance of new orthodontic composite arch wires (CAWs). The CAWs are made of a nickel and titanium alloy and stainless steel produced by laser welding, with copper serving as the interlayer. The copper interlayer can be damaged in the corrosive oral environment. The lysozyme coated arch wires were prepared using a liquid phase deposition with different concentrations of the enzyme. The corrosion behaviors of the CAWs and their antibacterial performance were examined: the composite arch wires coated with 20 g/L of lysozyme showed fewer corrosion pit and less corrosion depth relative to the uncoated CAWs. The concentration of 40 g/L of lysozyme was identified as the ideal future clinical application by the results observed against *S. aureus*.

#### 3.1.3. Biofilms

Biofilms are matrix-enclosed accumulations of microorganisms, such as bacteria, fungi, protozoa, and viruses. They are rarely composed of a single type of cells. The non-cellular components can be carbohydrates, proteins, lipids, lipoproteins, and lipopolysaccharides. The adhesion of microorganisms can be encountered in the food environment (food processing and packaging). Some of the nosocomial infections are caused by the use of biomedical devices on which the biofilm of *Staphylococci* bacteria grow. Frequently, the common surface cleaning procedures are not sufficient against mature biofilms. Biofilm structures cause the reduced response of bacteria to antibiotics and weakens the bactericidal actions of antimicrobial and sanitizing agents. Many efforts are focused on the identification of surfaces able to prevent the adhesion of microorganisms and, at the same time, exhibiting biocide properties.

In a 2004 U.S. Patent [123], the preparation method of a two-component composition of two anchor enzyme complexes was described. The first complex degrades the biofilm structures. For example, it contains alginate lyase to degrade the polysaccharide matrix in which *Pseudomonas aeruginosa* can grow and proliferate. The second anchor-enzyme complex contains the lysozyme necessary to lyse the bacteria within the biofilm. This composition can be utilized on implanted medical devices (catheters and cannulae) to control a variety of infections.

*Enterococcus faecalis* is a Gram-positive gastrointestinal commensal and a leading cause of nosocomial infections. *E. faecalis* infections are difficult to treat because it forms biofilms and is resistant to many antimicrobial agents. The effect of lysozyme (from chicken egg white and rhLys) on *E. faecalis* biofilm was studied in a 2019 patent and a kit with lysozyme was developed for treating such a bacterial infection [124].

In another case [125], with the aim to protect stainless steel surfaces, the lysozyme from a hen egg white and PEG were coated onto the surfaces. The surfaces coated with the enzyme displayed high antiadhesion activity toward *Listeria ivanovii* and a marked local biocide activity on *Micrococcus luteus*. 

**Figure 4:**
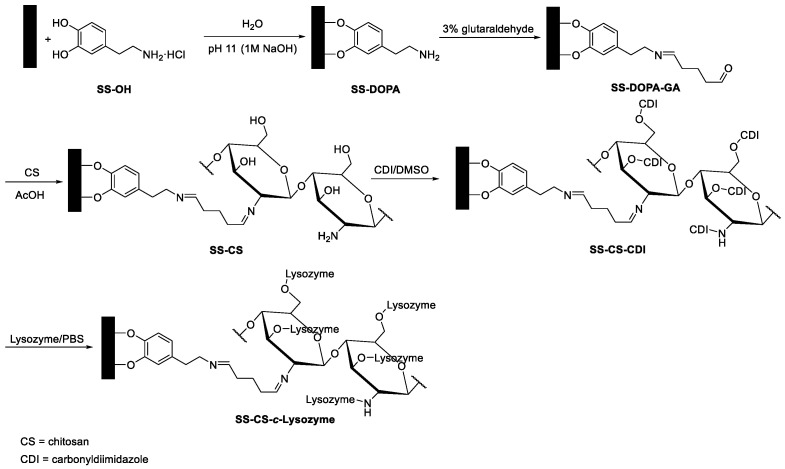
A schematic diagram illustrating the process of coupling dopamine, bifunctional cross-linker glutaraldehyde, chitosan, and lysozyme.

In another biofilm study, stainless steel surfaces were chosen as the starting material in order to obtain a material with a bactericidal function [126]. The surface was first activated by a biomimetic catechol (dopamine) anchor; the amino group of dopamine was treated with glutaraldehyde, a bifunctional linker. Chitosan was covalently immobilized by the reaction with glutaraldehyde. Finally, lysozyme (from the hen egg white) was conjugated to the chitosan (Figure 4). Chitosan is a well-known biopolymer endowed with antibacterial activities against bacteria, fungi, and algae [127]. The modified surface showed that the properties of both chitosan and lysozyme were preserved, and a substantial enhancement of the activity against bacterial adhesion and bactericidal efficiency against *S. aureus* were observed on the lysozyme-immobilized substrates relative to the chitosan-grafted substrates.

The immobilization of lysozyme on chitosan would offer a method for combating the biofilm-related contamination of stainless-steel implants and devices.

*Streptococcus pneumoniae* is a Gram-positive bacterium which causes many infections, ranging from the common and usually mild otitis and rhinosinusitis to invasive diseases, such as sepsis, pneumonia, and meningitis. Since it has become increasingly resistant to antibiotics, new antimicrobials have been developed, including the use of bacteriophages and some of their products. Two pneumococcal phage lysozymes (Cpl-1 and Cpl-7) were chosen with the aim to construct four dimeric proteins by shuffling their two functional domains and the linkers. The ability of these new enzymes to reduce pneumococcal biofilm formation and the killing activity against pneumococcal bacteria were studied in mice. One of them showed a greater bactericidal capacity than the natural Cpl-1, confirming the power of this new chimeric enzyme [128].

Another *Streptococcus*, *S. mutans*, causes the formation of a biofilm in the human oral cavity and tooth decay. A study was carried out to improve the activity of marine *Arthobacter oxydans* KQ11 dextranase mouthwash by the best combination of ZnSO_4_, lysozyme, citric acid, and chitosan. The optimized formula tested in marine dextranase mouthwash enhanced the inhibition of *S. mutans* [129].

Antibacterial photodynamic therapy is used for caries, endodontic disease, periodontitis, and peri-implantitis. This technique utilizes reactive oxygen species, such as singlet oxygen and free radicals via photosensitizers, to reduce bacterial infections. In order to overcome the narrow spectrum of excitation wavelengths to generate O_2_ of the commonly chosen photosensitizers, a novel Au nanocluster photosensitizer was proposed [130]. This novel photosensitizer is a lysozyme-gold nanocluster (AuNCs)–rose Bengal (RB) conjugate. This conjugate showed to effectively inhibit the growth of oral Gram-negative and Gram-positive bacteria when exposed to white light-emitting diode irradiation. The photoexcited Lys-AuNCs-RB successfully decreased the formation of *S. mutans* biofilm. 

The formation of biofilm around implants is the primary cause of infections in the mucosa and bone adjacent to the implant. The incorporation of antibacterial compounds into the coating of dental implants is an effective strategy to prevent biofilm formation. Lysozyme was immobilized on titanium surfaces forming an initial functional layer. This base was subsequently coated with silver nanoparticles, chitosan, and hyaluronic acid via a layer-by-layer, self-assembly technique. The obtained surface was examined by SEM and X-ray photoelectron spectroscopy. The inhibition of the biofilm formation and the antibacterial activity were evaluated against *S. aureus*. The results showed that the modified Ti surfaces were effective in preventing bacteria colonization for 14 days, until mucosa healing [131]. 

The same techniques were followed, starting from a metal laser sintered Ti implant primed with phase-transited lysozyme, loading minocycline into polyelectrolyte multilayers, obtained, in turn, by the self-assembly of negative charged hyaluronic acid and positively charged chitosan, natural polysaccharides, non-toxic, and biodegradable [132]. The obtained modified titanium exhibited a strong antibacterial effect against the oral bacteria *Streptococcus sanguis* and *Streptococcus gorgonii*, two pathogenic species in the biofilm formation process.

*Candida albicans* is a yeast present in the oral cavity that can incorporate that form into biofilms on denture surfaces, leading to, in some cases, denture stomatitis. Lysozyme present in saliva (1–57 μg/mL) contributes to the control of oral microflora. In 2017, an in vitro study [133] investigated the effects of lysozyme on *C. albicans* biofilm formation. The results showed that at a low concentration (<30 μg/mL), lysozyme reduced the attached biomass, whereas with concentrations of >300 μg/mL, a pro-biofilm effect was observed.

Among the substances suitable for use as a raw material, for the preparation of surfaces effective for inhibiting biofilm formation, polyhydroxyalkanoate [134] was chosen to form sheets, onto which lysozyme was loaded. The maximum loading was 16.1 μg enzyme per 9.5 mm^3^ disks. These sheets, endowed with an effective inhibition for biofilm formation, can find an application for the fabrication of wound dressing in anti-biofilm treatments.

To remove bacteria biofilm, new ways to deliver antimicrobial agents in a sufficiently high concentration are required. Liposomes are considered as an attractive carrier for drug delivery for their potential to fuse with phospholipid membranes. The spontaneous fusion, resulting in payload loss, can cause a problem. In 2017 [135], a Chinese team proposed a novel lysozyme preparation as a gentamicin carrier. The antibiofilm activity of lysozyme, associated liposomal gentamicin, was tested against *Pseudomonas aeruginosa*, a Gram-negative human pathogen. This microorganism causes chronic pulmonary infections and is generally employed as a model organism for the investigation of biofilm. The study demonstrated that the positively charged lysozyme stabilized the negative-charged liposomes, and the lysozyme-associated liposomal gentamycin was more effective against the biofilms built by Gram-positive (*S. aureus*) and Gram-negative bacteria than lysozyme or gentamycin alone.

The lysozyme activity, in combination with cefepime or ceftazidime against the *Pseudomonas aeruginosa* biofilm, was recently studied [136]. The results showed that 50 times of the minimum inhibition concentrations (MICs) of 2 cephalosporins, in presence of lysozyme (30 μg/mL), significantly inhibited the 24 h-old biofilm that was formed, when compared with individual antibiotic treatment. The highest inhibitory effect (a 49.3% reduction) was observed with the combination of cefepime and lysozyme. This method can be applied to the eradication of *P. aeruginosa* biofilms from catheters, ocular lenses, intravascular devices, and ventilator tubes.

This synergistic effect against *P. aeruginosa* was also observed when a combination of ciprofloxacin (at sub MIC of 1.56 μg/mL) with lysozyme was applied. Indeed, in the case of the combination, a 56 ± 0.6% biofilm eradication was obtained, whereas a 40 ± 0.5% eradication was observed with ciprofloxacin, alone [137].

The co-administration of an antibiotic, clindamycin, or metronidazole, and rhLys, improved both the efficiency of the antibiotics and the lysozyme-driven biofilm degradation. This cotreatment was applied in an in vitro study on the biofilm of *Gardnerella vaginalis*, the bacterium involved in vaginal infections. Often, the antibiotic treatments are not resolutive and the patients suffer from recurrent infections, probably due to the inability of the antibiotics to reach the bacteria embedded inside the biofilm. The bactericidal and biofilm degradation effects, tested in this in vitro study, were greater than when lysozyme or antibiotics was tested alone [138].

The application of nanotechnology can overcome the difficulty exhibited by most antibiotics to cross the barriers of biofilm. In this context, C-dots, a new class of carbon nanomaterials, were synthesized from papaya leaf extracts and encapsulated with lysozyme into chitosan nanocarrier forming C-dots carriers (CDCs) [139]. The antibacterial activity of CDC was tested on *B. subtilis* (Gram-positive) and ampicillin-resistant *E. coli* (Gram-negative). The biofilm inhibition was studied on pellicle of *B. subtilis*. The combination of C-dots and lysozyme delivered by chitosan nanocarriers, inhibited the growth of both Gram-positive and Gram-negative bacteria, but more efficiently against Gram-positive bacteria. In addition, CDCs showed the capability not only to inhibit the biofilm formation, but also to eradicate the established biofilm. The authors are investigating the antibiofilm activity of CDCs on the surface of medical implants.

#### 3.1.4. Oral Care

The antibacterial properties of lysozyme recently found applications also in the preparation of oral care solutions [140], stain-removing and whitening toothpaste [141], oral sprays [142], antibiomembrane remineralization material for root canal irrigation, and blocking treatment of dentin caries [143].

Recently, an oral cavity care spray containing lysozyme was proposed for dogs and cats [144].

A reduction of gingival inflammation was observed by the treatment of chronic periodontitis with a combination of vitamin C, vitamin E, lysozyme, and carbazochrome [145].

The antimicrobial activity of lysozyme against cariogenic microorganisms was evaluated in vitro. The bactericidal and bacteriostatic effects against *Streptococcus mutans* (68.5 mg/mL and 58.7 mg/mL, respectively) and *Lactobacillus casei* (50.3 mg/mL and 43.1 mg/mL, respectively) were quantified. The two microorganisms were not affected by lactoferrin, another saliva protein, or by the synergic use of lysozyme and lactoferrin [146].

The growth inhibition of *Streptococcus mutans*, by a mixture of casein phosphoprotein/amorphous calcium phosphate, lysozyme, lactoferrin, and lactoperoxidase, was evaluated in a caries model. The results showed that the progression of carious lesions was prevented [147].

The role of salivary proteins, including lysozyme, in cariology, was reviewed in 2004 by a Dutch team [148].

#### 3.1.5. Respiratory Disorders

The administration of lysozyme by aerosol for the treatment of respiratory disorders, was suggested in a 2001 U.S. Patent [149]. Moreover, according to the same authors, it can be used as vehicle for the intratracheal administration of drugs to the lungs [150].

Aerosolized rhLys was used as an alternative to conventional antibiotics, to treat induced pneumonia in hamsters. The animals were inoculated with *P. aeruginosa*, then treated with a 1% solution of rhLys in water (2 h × 3 days). The treatment significantly reduced the parameters related to pneumonia, such as the bacterial colony-forming in the whole lung and bronchoalveolar lavage fluid (BALF), and the total BALF leukocytes. [151].

*P. aeruginosa* is the major airway pathogen in patients with cystic fibrosis. A fusion protein, composed of lysozyme and surfactant protein B, was proposed for the prophylaxis or the therapeutic strategy for suppressing the bacterial colonization of the airways in cystic fibrosis patients. The efficacy of the fusion protein was studied in transgenic mice, expressed with this combination of lysozyme and surfactant protein B, after intra-tracheal injections with *P. aeruginosa*. The bacterial clearance was enhanced from six- to thirty-fold in mice from different transgenic lines, compared to the values observed in wild-type controls [152].

The occurrence of long-term infections in cystic fibrosis is also due to the inactivation of native airway defense. The inflammatory response to the infections causes the formation of high concentrations of negatively charged polymers, which bind and sequester natural positively charged antimicrobial and antibacterial proteins. The reduction of this sequestration by means of the oppositely charged polymers in the airways was the object of a 2007 patent. A human lysozyme was charge-modified by reducing the net charge to a less positive charge level. The charge-modified lysozyme was composed by a first segment having the amino acid sequence of a natural lysozyme and a second segment having from two to ten negatively charged amino acids. A composition of the charge-modified lysozyme and a cationic lipid decreased the antimicrobial sequestration, which reduced the lysozyme binding to actin or DNA, two polyelectrolytes naturally produced from the inflammatory response [153].

The actin-lysozyme complexes formed in cystic fibrosis disease were studied in 2006 [154] and, more recently, in 2016 [155], by synchrotron small-angle X-ray scattering and molecular dynamic simulations, in order to establish their structure, stability and behavior in the presence of the physiological salt concentration of airways.

#### 3.1.6. Gastrointestinal Tract Diseases

Patients who take drugs, such as proton pump inhibitors to reduce or treat gastric hyperacidity, have a stomach pH value of around 5. This value allows *Enterobacteriaceae*, for example, certain strains of *E. coli*, to pass through the degraded gastric barrier.

Compositions of N-acetylcysteine and microencapsulated gastro-protected lysozymes with probiotic bacteria were proposed in a 2014 patent, to restore the stomach’s barrier effect, avoiding the suspension of the pharmacological treatment that would expose gastric and esophageal mucosa to the damage caused by gastric juices and the risks of infections. In the patent, the quantity of N-acetylcysteine, lysozyme, and the list of possible probiotic bacteria were reported. The proposed composition comprised of strains capable of producing bacteriocins, and it is a useful adjuvant in the treatment of *Helicobacter pylori* [156].

The lysozyme of the microbial source was also proposed for suppressing the growth and/or intestinal colonization of bacterial pathogens in the gastrointestinal tract, and in the treatment of irritable bowel syndrome or inflammatory bowel disease (Crohn’s disease and ulcerative colitis). The composition can be administered in the form of foods or dietary supplements [157].

A new lysozyme composition containing natural biological correctors to prevent iron deficiency, calcium deficiency, and gastric disorders was developed in Kazakhstan. In addition to lysozyme, this food supplement contains herbal substances, briar pectin, and dry whey, a calcium-containing product [158].

The genus *Salmonella* is known as an agent that causes diarrheal disease in humans. *Salmonella* O48 strains cause enteritis in humans, especially children, and in animals. They are sensitive to the bactericidal action of normal bovine serum and normal human serum. The serum complement system consists of at least 35 proteins and it is a part of the adaptive and innate immune system. The activation of complement is achieved through three different pathways. In order to establish the role of lysozyme in the mechanism of the activation of bovine and human complement, a study was carried out in 2009 [159]. Lysozyme was removed from serum by adsorption on montmorillonite. The results showed that the most efficient killing of *Salmonella* O48 was obtained when all the components of bovine or human serum cooperated, and that the lysozyme was an obligatory factor in the bactericidal action of the components of normal bovine serum and human serum, taking place on the activation mechanism.

#### 3.1.7. Ophthalmic Applications

Lysozyme is one of the major components in human tears, where it plays the role of natural defense against ocular infections.

In the situations of dry eye or inflammation (for example ulcerative herpetic keratitis), tears are not being produced in the normal quantity or quality, or are not produced at all. For this reason, many ophthalmic solutions contain the hen egg white lysozyme. However, its use may cause allergic reactions. In a 2008 U.S. Patent [160], rhLys, instead of the hen egg white lysozyme, was used as the agent that afforded protection from microorganisms, not only for the ophthalmic solutions themselves but also of the eyes to which they were applied. The hLys containing solutions can also be used to condition and clean contact lenses.

The posterior capsule opacification (secondary cataract) is a complication that occurs from having cataract surgery, due to the immune response or the adhesion and reproduction of residual human lens epithelial cells (HLECs) on the posterior capsule. Infectious endophthalmitis is another complication, due to bacterial colonization (coagulase-negative *Staphylococci* and *S. aureus*) and biofilm formation. Hyaluronic acid-lysozyme composite coating was covalently grafted onto the surface of intraocular lenses (poly-methylmethacrylate). The presence of hyaluronic acid, due to its hydrophilic properties, reduced the adherence of *S. aureus* and HLECs on the poly-methylmethacrylate, whereas lysozyme (from hen eggs white) exerted its own bactericidal activity against *S. aureus*, as shown in the in vitro test. The immobilized lysozyme and hyaluronic acid conferred either anti-adhesive and antibacterial properties to the surface of intraocular lenses, making them potentially useful for the prevention of endophthalmitis and posterior capsule opacification [161].

#### 3.1.8. Otitis and Sinusitis

Lysozyme is expressed in the tube tympanic of mammals, playing an important role in protecting the middle ear and Eustachian tube.

Otitis media, or middle ear infection, is a common pediatrics disease and the increase in the resistance to antibiotics among the otitis media pathogens, such as *Streptococcus pneumoniae*, prompted the development of non-antibiotic approaches. 

The effects of lysozyme on middle ear infections were studied on mice, in 2008 [162]. Mice have two lysozyme genes: lysozyme M expressed in macrophage, bone narrow, and lung tissue, and lysozyme P, mainly expressed in the small intestine and at much lower levels in other cells/tissues. Wild-type mice and lysozyme M^−/−^ mice were compared to verify if the lysozyme M depletion increased their susceptibility to pneumococcal otitis. A deficiency in lysozyme M led to an increased susceptibility to middle ear infection with *S. pneumoniae* and resulted in severe middle ear inflammation. The authors suggested that exogeneous lysozyme can be used as an adjuvant therapeutic agent for otitis media. Lysozyme and other antimicrobial proteins and peptides were investigated by the same research team, and their use was suggested for the treatment of otitis media and paranasal sinusitis [124,163]. 

#### 3.1.9. Anti-Inflammatory Effects

In addition to the antibacterial enzymatic activity, lysozyme displays anti-inflammatory effects: it is up-regulated in the gastrointestinal tract of patients affected by chronic inflammation as Bannet’s esophagitis, chronic gastritis, coeliac disease, and colitis. The supplementation of the exogenous hen egg white lysozyme has a significant anti-inflammatory effect in the treatment of inflammatory bowel disease [164]. 

The anti-inflammatory effects of lysozyme against an extracellular mediator of vascular inflammatory disease, sepsis, was described in 2015 [165].

More recently, in 2019 [166], the anti-inflammatory effect of lysozyme was studied, investigating the perturbation of gene expression profiles induced by the in vitro supplementation of lysozyme (15 μg/mL for 1 h or for 24 h) in a monocyte cell line, a type of cell that interacts with lysozyme. The proteins in the cell line were identified and quantified by LC-MS/MS. The results showed that the anti-inflammatory activity of lysozyme was due to the mechanism of gene regulation involving the proteins of the inflammatory pathway, such as tumor necrosis factor alpha (TNF-α) and interleukin 1 beta (IL-1β).

### 3.2. Lysozyme as Food Preservative 

Food spoilage is the result of chemical, physical, or microbiological modifications that renders the food unacceptable. Microbial food spoilage occurs due to the growth of microorganisms (bacteria, fungi, and yeasts) in or on the food, and it is also the cause of most food-borne illnesses.

To overcome these undesired effects, manufactures and producers employ multiple strategies for food preservation, which include chemical, biological, and physical treatments. Lysozyme, in place of traditional antibiotics, can be utilized to preserve food and beverages in much the same manner, with reduced side effects practically limited to triggering adverse allergic reactions in susceptible individuals. For this reason, its presence in foods and beverages in many countries required the inclusion of egg allergens in labeling, when present in the final product (see, for example, Regulation (EU) No 1169/2011 of the European Parliament and of the Council of 25 September 2011).

The use of lysozyme as a food additive is permitted in cheese, since it exerts an inhibitory effect against the growth of lactic acid bacteria involved in curd acidification and cheese ripening [167]. It also inhibits the growth of *Clostridium tyrobutyricum* that causes late blowing in hard and semi-hard cheese. In wine making, a maximum of 500 mg/L of lysozyme has been permitted, since 1996 (resolution OENO 10/97), because it helps to control malolactic fermentation by limiting lactic acid bacteria proliferation [168]. For unpasteurized beer, the concentration of 300 mg/L of lysozyme can reduce lactic acid beer spoilage bacteria, such as *Pediococcus inopinatus*, *Lactobacillus brevis*, *Lactobacillus brevisimilis*, and *Lactobacillus lindneri* [169].

To overcome the allergenic risks, due to the presence of lysozyme that can remain in food and beverages, different strategies were studied, including the immobilization through the Maillard reaction on different polysaccharides, to obtain films endowed with antimicrobial properties that can extend the shelf life of wrapped foods. For example, a bilayer edible film, based on chitosan and sodium alginate and incorporated with lysozyme, was demonstrated to be effective against fish spoilage bacteria, *Pseudomonas fluorescens* and *Shewanella putrefaciens* [170]; a lysozyme-chitosan composite film presented antimicrobial functions against both Gram-positive and Gram-negative representative bacteria (*E. coli* and *S. faecalis*) [171]; a lysozyme–dextran conjugate was effective against *S. aureus* and *E. coli* in a natural food system (cheese curd) [172]; a lysozyme–sodium alginate edible film was tested on two Gram-positive bacteria (i.e., *Listeria innocua* and *Kocuria rhizophila*) giving positive results [173]; lysozyme integrated into chitosan nanoparticles showed improved antibacterial activity against *E. coli* and *B. subtilis* [132,174]; and a chitosan composite film containing lysozyme–rectorite showed reduced mechanical properties (−28%) in regard to the film containing only chitosan and enhanced antibacterial properties on *E. coli* and *S. aureus* [175]. With a different approach on foods already containing egg derivatives, lysozyme immobilized on a polysaccharide was added to the food, in order to improve its functional properties and to extend the shelf life, such as in the case of mayonnaise treated with lysozyme immobilized on Arabic gum [176]. The attempt to reduce the allergenicity of lysozyme-treated wine using a thermal or an enzymatic treatment was carried out without producing a convincing outcome [168].

Since lysozyme exhibited weak inhibitory effects against Gram-negative bacteria, when used alone, the practical application of free lysozyme as a food preservative was quite limited and many methods used to enhance the susceptibility of Gram-negative bacteria were attempted, namely the combined use with cinnamaldehyde [177]; acidic electrolyzed oxidizing water [178]; heat treatment and hydrogen peroxide associated with a packaging in a controlled atmosphere [179]; high hydrostatic pressure [180]; disodium ethylenediaminetetraacetate salt and ethylenediaminetetraacetic acid [181,182]; chitooligosaccharides [183]; chitosan-organic rectorite composites and negatively charged sodium alginate film-coated cellulose acetate mats [184]; EDTA in starch-based active food packaging film [185]; enterocin AS-48 [186]; and pomegranate peel extract [187]. The relevant data on the synergic uses of these substances with lysozyme are summarized in Table 1.

### 3.3. Feed Uses of Lysozyme 

Antibiotics have been used for many years in animal production. They are included in animal feeds at subtherapeutic levels to improve growth rate and feed efficiency, for disease prophylaxis, and at higher doses, for disease therapy. Even though antibiotics have been used by growers as effective supplements, their use, nowadays, as feed additives in animal production is strongly limited or banned on the basis of the “precautionary principle” that resistance selected in animals can be transmitted to humans to the detriment of their health [189]. Historically, the first banned antibiotic for animal use was avoparcin, in 1997, followed by bacitracin, spiramycin, tylosin, and virginiamycin, in 1999 [190]. To manage this problem, it is necessary to improve the biosecurity by avoiding, if possible, the use of traditional antibiotics and looking for safer alternatives, such as dietary supplements, including probiotics, prebiotics, and natural antibiotics, including lysozyme, which are adequate for animal production.

Several studies explored the advantages of using lysozyme alone, as a dimer or in association with other proteins, antibiotics, or EDTA, for feed uses in poultry, pigs, cows, fish, and rabbits, and for crop protection. 

These studies investigated the influence of the dietary supplementation of lysozyme on growth performance (body weight (BW), average daily gain (ADG), and daily food intake (ADFI)) and the changes of microbiota in treated animals.

#### 3.3.1. Poultry

In a 2020 study, the influence of dietary supplementation with some antibiotic alternatives, including lysozyme, on the growth performance, intestinal barrier, and immunity of lipopolysaccharide-challenged chicks was investigated. The evaluation of lysozyme as an alternative antibiotic aureomycin in growing yellow-feathered chicks was carried out. The results showed that the dietary addition of lysozyme (500 mg/Kg) had a comparable effect to the antibiotic treatment on the jejunal barrier and on the immunity of yellow-feathered chicks aged from 4–24 days. From a biochemical point of view, the lysozyme treatment reduced diamine oxidase activity and the inflammatory mediators in plasma, jejunal mucosa, spleen, and thymus; increased the content of immunoglobulin in plasma and jejunal mucosa; and decreased the gene expression of inducible nitric oxide synthase and cyclooxygenase 2 in jejunal mucosa. This lysozyme treatment was, however, ineffective to retrieve the normal BW, ADG, and ADFI of the chicks after liposaccharide treatment [191]. In another paper, the chicks were fed a starter (1–21 days) and a grower (22–42 days) diet supplemented with 0 (control), 40, 100, or 200 ppm lysozyme, or 400 ppm flavomycin as an antibiotic control for 6 weeks. Lysozyme administration did not contribute significantly to the growth of the broiler chickens. No significant differences in the diversity and composition of the bacterial and fungal flora in the cecal microbiota of chickens in the different diet groups were found. However, lysozyme supplementation led to a significant enrichment of genes involved in the synthesis/degradation of bacterial outer membranes and cell walls, cross-cell substrate transport, and carbohydrate metabolic processes, thus possibly promoting the cecal microbiota carbon and energy metabolism [192]. 

Necrotic enteritis is a worldwide poultry disease caused by the overgrowth of *Clostridium perfringens* in the small intestine. The addition of exogenous lysozyme (40 mg lysozyme/kg) to the diet of chickens significantly reduced the concentration of *C. perfringens* in the ileum, and the intestinal lesion scores inhibited the overgrowth of *E. coli* and *Lactobacillus* in the ileum and intestinal bacteria translocation to the spleen, whilst improved intestinal lysozyme activity in the duodenum and the feed conversion ratio of chickens. These findings suggest that the exogenous lysozyme can decrease *C. perfringens* colonization and improve the intestinal barrier in chickens [193]. In another trial, the effect of a feed of 100 ppm lysozyme with EDTA (1/4 *w*/*w*) on the growth performance and intestinal microbiota of broiler chickens in each period of the growth cycle (total 35 days) was evaluated. The inclusion of virginiamycin or lysozyme in the broiler diets, at different periods during their growth, had no effects on their daily weight gain (DWG), feed consumption, or feed conversion rate (FCR), in both of the new litter and used litter trials. Moreover, the number of aerobic bacteria, anaerobic bacteria, coliforms, *E.coli*, lactic acid bacteria, and *C. perfringens* in the ileum was not influenced by the inclusion of the antibiotic or lysozyme in the diets [194]. 

In poultry and swine production, the administration of lysozyme through water or feed in association with nisin, albumen, and sequestering agents, such as EDTA, citric acid, or chitosane, was effective to inhibit the growth, diseases, and epidemiology effects caused by *C. perfringens*, *E. coli*, *Salmonella typhimurium*, and *Salmonella Mbandaka* in the gut of livestock. Therefore, it is useful in the treatment of necrotic enteritis, *C. perfringens* enteritis, and diarrheal disease [195]. A recent patent application claimed a composition containing rhLys (10–20%), *Bacillus coagulans* spore powder (20–30%), *Lactobacillus plantarum* (5–15%), fructooligosaccharide (5–15%), and auxiliary materials as a poultry and livestock feed additive. This composition, according to the authors, can replace antibiotics to be mixed with feed or water, as a livestock growth promoter; it can also be used to prevent and treat common animal diseases and improve immunity and survival rates [196].

#### 3.3.2. Pigs

A review on lysozyme as an alternative to growth promoting antibiotics in swine production was published in 2015 [197] and, more recently, in 2019. In 2021, two meta-analyses focused on the evaluation of the antibacterial activity and the impact on the performance of weaned pigs of potential dietary feed additives, including lysozyme, were published [198,199]. The use of lysozyme, as a food additive at a dosage comprises between 0.01% and 0.2%, showed an ADG of 28 g higher, with respect to the negative control group (normal feed), while no significant difference with the positive control group was observed (treated with antibiotics). A feed reduced by 52 g was required in the treatment group, compared to the negative control group, to gain 1 kg of bodyweight, while no significant difference with the positive control group was observed. 

The results of a clinical trial carried out to investigate the response of piglets receiving a water-soluble lysozyme/EDTA mixture (known by the trade name of Entegard^®^, EG, Neova Technology Inc., Abbotsford, BC, Canada), delivered in the drinking water at concentrations of 0.1% and 0.2%, after an oral challenge with enterotoxigenic *E. coli* (ETEC), compared with a control with no additives or antibiotics (2.5 g/kg of an antibiotic feed with chlortetracycline, sulfamethazine, and penicillin), confirmed that this treatment did not influence the growth performance throughout the study. However, better intestinal growth and development, as well as decreased ETEC counts in the intestinal mucosa and serum proinflammatory cytokines, suggested that lysozyme/EDTA could maintain gut health and function in piglets commensurate with antibiotics. In this paper, it was observed that, in the largest tested dose, lysozyme/EDTA seemed to have a dramatic effect on proinflammatory cytokines, but had a minimal or no effect on the other response criteria [200]. The results of a similar test carried out on 10-day-old pigs that included a feed supplement with lysozyme/EDTA (100 mg/L water solution), in comparison with traditional antibiotics (i.e., neomycin and oxytetracycline, 16 mg/kg of diet), to evaluate the growth performance and small intestinal morphology after a *Campylobacter* infection confirmed that lysozyme/EDTA was a suitable alternative to antibiotics for 10-day-old pigs consuming manufactured liquid diets. More significantly, this work confirmed that pigs gained a weight of 334 ± 12 g/day from 0 to 7 days of treatment, regardless of dietary treatment. However, from 7 to 14 days, pigs fed with the lysozyme/EDTA and antibiotic treated diets gained BW at a greater rate, compared with pigs fed with the control diet, which resulted in an overall faster ADG from 0 to 14 d (402 ± 12 and 422 ± 14 g/day vs. 364 ± 14 g/day, respectively) [201]. The outcome that lysozyme improved the growth rate and can be effective for pathogen control, when fed in nursery diet, was confirmed by other papers in which lysozyme was administered in the diet at a concentration of 100–120 mg/kg [202,203,204,205]. Oliver et al. observed changes in small intestinal morphology (in the jejunum, the villi height to crypt depth ratio increased by 72%) in nursery pigs consuming lysozyme. It remains to be seen whether the intestinal changes were mechanistically responsible for the improved feed conversion. Further studies confirmed the above assumptions and, in addition, showed that lysozyme, as well as antibiotics, increased protein deposition at the expense of lipid accretion, since the immune response in pigs redirected nutrients away from growth and toward the immune system and ameliorated the effects of a chronic immune challenge [206]. 

A study on the variations in the gut microbiota of sows, serum immunity and breast milk metabolite profiles mediated by lysozyme supplementation was published, in 2018 [207]. Sows that received 1.0 kg/t of lysozyme treatment showed significant variation that was determined by the high-throughput sequencing of the microbial 16S rRNA genes, in microbial diversity: *Spirochaetes*, *Euryarchaeota* and *Actinobacteria* significantly increased, while *Firmicutes* showed a remarkable reduction compared with control. Different to the above cited works, a paper published in 2021 focused on the evaluation of the efficacy of lysozyme on the growth performance, nutrient digestibility, excreta microflora population, and blood profiles of weanling pigs under *E. coli* challenge, but showed unsuccessful results since lysozyme did not improve the growth performance (ADG) after inoculation [208]. The authors suggest that the different outcome of their results can be due to the different sources of lysozyme, different species of *E. coli*, or to the presence of a direct *E. coli* K88 (the strain they used) challenge. 

In 2018, a paper was published evaluating the protective effect against the enterotoxigenic *E. coli* (ETEC) infection of neonatal piglets fed with milk from transgenic pigs that express high levels of rhLys (about 1300.7 ± 126.7 mg/L), compared to neonatal piglets fed with milk from wild-type sows. The observed protective effect of rhLys (i.e., facilitated faster recovery from infection and decreased mortality and morbidity following an ETEC oral inoculation or infection acquired by contact exposure) was associated with the enrichment of intestinal bacteria, such as *Lactobacillus*, that improve gut health and the enhancement of the mucosal IgA response to the ETEC-induced diarrhea [209]. The simultaneous expression of hLys and human lactoferrin (hLF) in bi-transgenic pigs, and the evaluation of the milk’s antibacterial ability, were evaluated. The results confirmed that pig milk containing hLF and hLys (at 6.5 g/L and 1.1 mg/L, respectively) had synergistic antimicrobial activity, as determined using the *M. lysodeikticus* test [210].

#### 3.3.3. Cows

The utilization of lysozyme as an alternative antibiotic in the veterinary field was explored in several patents and papers, particularly for the treatment/prevention of mastitis and in combination with other substances as antiviral to treat the bovine viral diarrhea virus (BVDV). In 2008, a patent application about a topical composition containing lysozyme, serratiopeptidase, *Oscimum sanctum*, and *Azadirechta*, for the prevention and/or treatment of mastitis and metritis in mammals, was filed [211]. In 2021, another biological disinfectant preparation for preventing mastitis in dairy cows, which included bactericidal effective amounts of lysostaphin (0.1–10%), lysozyme (1–40%), and other auxiliary components, was patent protected [212]. A recent study confirmed that the effectiveness of antibiotic treatment combined with lysozyme dimer was 58.3%, and comparable with other methods of supportive therapy. In this study, the use of lysozyme dimer (5.0/10 mL; Lydium-KLP, Nika Health Products Sp. z o o, Śmigiel, Poland) at a dose of 0.02 mg/kg increased the therapeutic efficiency by 6.7%, compared with the control mastitis group. However, this difference was considered by the same authors as not statistically significant [213]. As an alternative approach, a gene therapy strategy for treating bovine mastitis was developed in 2006: a new mammary-specific vector containing hLys, cDNA, and the kanamycin resistance gene for intramammary expression and clinical studies was constructed. The clinical studies showed that the injection of the diseased quarters twice with the p215C3LYZ vector had an overt therapeutic effect on bovine mastitis (in particular, clinical mastitis), including the disappearance of symptoms, a significant improvement in the California mastitis test score, and a reasonably high microbiological cure rate [214].

In another paper, the antibacterial efficacy of an experimental formula containing 15% condensed donkey milk (lysozyme content 825 mg/L) for washing cows’ udders before milking, was evaluated. The antimicrobial activity of condensed milk was firstly evaluated in vitro, using the disk diffusion method on the following microorganisms: *Bacillus megaterium*, *Bacillus mojavensis*, *Clavibacter michiganensis*, and *Clostridium tyrobutyricum*. These tests were compared with the effects of two antibiotics, ampicillin (100 mg/mL) and kanamycin (50 mg/mL), and a commercial pre-dipping formula, showing that the inhibitory activity of lysozyme from donkey milk on all the considered microorganisms was higher than that of the commercial product and similar to that of the two antibiotics [215]. Bovine viral diarrhea virus (BVDV), an enveloped, single-stranded, positive-sense RNA virus from the *Flaviviridae* family, is a globally distributed bovine pathogen. In 2019, the antiviral effect of naturally occurring proteins and peptides, such as bovine lactoferrin, chicken egg lysozyme, and nisin from *Lactococcus lactis*, used both individually and in combination, was evaluated in vitro, against the cytopathic NADL strain of BVDV. The study demonstrated that nisin and lysozyme were effective at later stages of the infection, and the intensity of their effect did not diminish with time. Nisin + lactoferrin and lysozyme + lactoferrin combinations demonstrated stronger antiviral effects than the single substances [216]. 

#### 3.3.4. Fish

In aquaculture, the accumulation of antibiotics resulted in the development of resistance among bacterial pathogens, and the possibility to utilize lysozyme instead of traditional antibiotics is an active and growing research field. In a 2015 study, a novel antimicrobial peptide, derived from the goose type lysozyme (LyzG), was identified from the cDNA library of freshwater fish, *Channa striatus* (Cs). The identified lysozyme cDNA contains 585 nucleotides, which encodes a protein of 194 amino acids. CsLyzG was closely related to *Siniperca chuatsi* with 92.8% homology. Two antimicrobial peptides, IK12 and TS10, were identified from CsLyzG and synthesized. The antibiogram showed that IK12 was active against *Salmonella enterica* and induced a loss of cell viability in the bacterial pathogen [217]. A 2016 patent application protected a feed composition for aquatic animals, especially for cold water fish (for instance, salmon, bream, and bass) and for warm water fish (for instance, carp, tilapia, and catfish) containing natural active substances (alpha-pinene, alpha-terpineol, cinnamaldehyde, dihydroeugenol, eugenol, meta-cresol, and terpinolene) in combination with a polypeptide having lysozyme activity. The polypeptides having lysozyme activity were isolated from fungus phylum *Ascomycota* and subphylum *Pezizomycotina*, and the amino acid sequence was identified. It was observed that this feed additive protected fish against infections caused by pathogenic microorganisms, Gram-positive microorganisms in particular, and to increase the feed conversion rate (FCR) and ADG [218]. In another study, the potential effects of dietary garlic supplementation on the health status of common carp (*Cyprinus carpio*) exposed to ambient ammonia toxicity was investigated. Indeed, high stocking density, in addition to un-eaten feed that decomposes in water, can lead to the accumulation of organic matter and toxic inorganic nitrogen, which can reach an unsafe level and threaten fish health. In this protocol, the fish were fed with either 0 (control), 0.5, 1, or 1.5% levels of garlic for 35 days, then challenged with 0.5 mg/L of ambient unionized ammonia-nitrogen for 3 h. The results of this study showed that garlic administration mitigated the adverse effects of ammonia exposure characterized by improving the antioxidant activities and decreasing plasma malondialdehyde, plasma glucose, and cortisol levels, and alanine transaminase; alkaline phosphatase; aspartate transaminase; and glutathione peroxidase activities. Moreover, it significantly increased plasma catalase, lysozyme, bactericidal activities, and Ig levels, but had no effect on the plasma superoxide dismutase activity [219]. The prospective use of jaggery (a concentrated product of cane juice) as a potential carbon source and its influence on water quality, growth performance, innate immunity, serum bactericidal capacity, and disease resistance to *Aeromonas hydrophila* was investigated in *Oreochromis niloticus*. It was demonstrated that the use of this carbon source had a pronounced effect on hematological and growth performance parameters in comparison with the control. Moreover, serum antioxidants, lysozyme, protease, antiprotease, and bactericidal capacity were significantly increased in the treated groups in a dose-dependent manner [220].

#### 3.3.5. Rabbits

A comparative study on the effects of dietary hen egg lysozyme (100 mg/kg) and/or zinc bacitracin antibiotics (ZnBs) on the productivity and health conditions of rabbits was carried out in 2021. After eight weeks, the final weight and body weight gain of rabbits that were fed dietary lysozyme and ZnB additives were meaningfully increased and the feed conversion ratio was markedly decreased. In addition to the enhancement of the growth performance, the rabbits treated with lysozyme and those treated with ZnB showed significantly lower populations of *Clostridium* spp. and *E. coli* compared with the control; whereas, the counts of *Lactobacillus* and total bacteria were meaningfully increased in the intestines of rabbits treated with lysozyme and ZnB. The levels of total protein, globulin, creatinine, urea, ALT, and AST, and the gene expressions of the liver superoxide dismutase and glutathione peroxidase of the treated rabbits were also reported [221]. 

### 3.4. Crop Protection

Lysozymes are naturally present in various plants in farm animal materials used for food and feed preparation, and for the prevention and treatment of some microbial infections, including the possibility to substitute conventional bactericides and biopesticides for crop protection. Using recombinant DNA technology, the microbial gene coding for the bactericidal T4 lysozyme (endo acetylmuramidase) was integrated into the genome of potato plants. Laboratory and field tests demonstrated the expression of the gene during plant growth and a markedly decreased infection of plants and tubers by the phytopathogenic bacterium *Erwinia carotovora* ssp. *atroseptica*. Environmental testing did not indicate any adverse effects of the transgenic plants on the soil microbiota, including plant-beneficial bacteria [222]. 

*Lysobacter enzymogenes* LE16 broth culture unheated self-digestive solution, or heated at 100 °C for 30 min, or stored for 12 months at room temperature, were prepared to study their potential as biocontrol agents. This bacterium produced protease, phosphatase, lysozyme, and siderophores in pure culture, as well as 12 secondary metabolites, including novel antibiotics lysobactin, WAP-8294A2, and mupirocin determined and based on the antiSMASH 5.0.0 BLAST database. A poison plate assay was carried out to test the antagonistic activities of these solutions against *S. aureus*, *P. syringae pv. tabaci*, and numerous plant pathogenic fungi, and oomycetes, including *Colletotrichum gloeosporioides*; *Penicillium italicum*; *Alternaria alternate*; *Rhizoctonia solani*; *Didymella bryoniae*; *Sclerotinia sclerotiorum*; *Phytophthora nicotianae*; and *Phytophthora capsici*. These experiments showed that the unheated self-digested *Lysobacter enzymogenes* LE16 broth culture was highly effective in controlling pepper blight disease, which is caused by *P. capsici*. Compared with pathogen-only inoculation, the application of unheated self-digestive solutions of *L. enzymogenes* LE16 to the soil, in preventive or curative treatments, significantly reduced the disease incidence and the index with a relatively high control efficacy of 86–93% [223].

In a study conducted in 2006, the effect of genetically modified (GM) potatoes producing anti-bacterial agents was investigated, such as cecropin/attacin and T4 lysozyme on the diversity and functional abilities of bacteria colonizing the intercellular spaces and vascular tissues (endosphere) of potato plants. These GM potatoes can offer effective future pathogen control strategies, particularly for the treatment of blackleg and soft rot disease of potatoes (*Solanum tuberosum* L.), mainly caused by the bacterial pathogen *Erwinia carotovora* ssp. *atrospetica* (Eca). In order to compare GM-related variations with impacts caused by changing environmental conditions, potatoes were cultivated in two different soil types, and challenged with the pathogen Eca. The endophytic diversity was assessed by 16S rRNA-based terminal-restriction fragment length polymorphism (T-RFLP) analysis. The result of this study confirmed that the impact of both genetic modification types was minor or comparable with the variations caused by plant genotype, vegetation stage, pathogen exposure, and soil type [224].

The control of fire blight, whose causal pathogen is the Gram-negative bacterium *E. amylovora*, has been mainly attempted by spraying the antibiotics streptomycin and oxolinic acid. Moreover, copper sprays or chemical resistance inducers, such as prohexadione Ca (Apogee) can be alternatively used. Alternative biological control was also tested using antagonistic bacteria *(P. fluorescens* strain A506), bacteriophages; yeasts were also used with some success to interfere with *E. amylovora* populations. In a 2006 paper, a viral lysozyme from the *E. amylovora* phage φEa1h affecting Gram-negative bacteria, such as the fire blight pathogen, was characterized. A lysozyme gene from the *E. amylovora* phage φEa1h was cloned and expressed in *E. coli*, and the protein activity compared with lysozyme from the *E. coli* phage T4. φEa1h lysozyme lysates strongly affected *E. amylovora* cells. The protein displayed enzymatic and antibacterial activities. Pear slices soaked in φEa1h lysozyme-containing cell lysates showed reduced fire blight symptoms after inoculation with *E. amylovora* [225]. An alternative approach to treat the infection by *E. amylovora* using a combination of lysozyme and of a synthetic peptide derivative was described in a paper published in 2017. BP100, a linear undecapeptide (KKLFKKILKYL-NH_2_), has been reported to be effective against *E. amylovora* infections in apple and pear flowers. However, peptide concentrations necessary for the control of fire blight disease were 10 to 50 times higher than the minimal inhibitory concentration (MIC). This decrease in the activity in plants has been attributed to the inactivation by certain plant compounds or structures, or to their degradation by proteases from plant tissues or epiphytic microorganisms. The combination of BP100 with lysozyme produces a synergistic effect. It was observed that while BP100 or lysozyme alone, at the concentrations tested, were not active, at 1.0 μM BP100 the effect of the addition of lysozyme was only observed at 0.5 mg/mL, and at 2.5 μM the effect on the growth inhibition was significant in all lysozyme concentrations. The significant increase in the antimicrobial activity against *E. amylovora* was associated to the increase in cell membrane damage and to the reduction of cell metabolism. The combination of BP100 with lysozyme reduced the time required to achieve cell death and the MIC, and increased the activity of BP100 in the presence of leaf extracts, even when the peptide was applied at low doses [226]. 

## 4. Synergistic Activity of Lysozyme

A synergistic interaction is defined as the observation of a combination effect that is greater than the sum of the effects of two independently administered agents, whereas additive interactions are when the combined effect is equal to the sum of the individual effects.

### 4.1. Antimicrobial Peptides (AMPs)

After the discovery of lysozyme, various types of molecules with antibiotic activity have been isolated from animals, insects, plants, and bacteria, among those the AMPs play an important role. The mammalian innate AMPs are essential for the protection of skin and other organs, but they also control physiological functions, such as inflammation, angiogenesis, and wound healing [227].

These peptides are defined as antimicrobial since they have abroad spectra of activity, including the ability to kill Gram-negative and Gram-positive bacteria, fungi, parasites, cancer cells, and even viruses, such as HIV and the herpes simplex virus. These peptides are stored in granules of phagocytic cells and are expressed and released at the epithelial surface and in a site of inflammation. Hancock and Scott, in two reviews published in 2000 [228,229], described how they can present the synergy with lysozyme disrupting the outer membrane that is the barrier that excludes lysozyme from the peptidoglycan. Most of the AMPs are cationic and they can adopt an amphiphilic conformation. As a result of these features, they can interact with the negatively charged compounds on the surface of bacterial cells and insert into the lipid bilayers.

The human lysozyme was reported by Hancock to show a good synergy with the AMP protegrin against *Enterococcus faecalis*: indeed the MICs of protegrin and lysozyme were lowered from 16 and 6 μg/mL, when used alone, to 4 and 0.39 μg/mL, respectively, in combination [230]. The combination of human lysozyme with six different AMPs against *P. aeruginosa* was recently examined and the authors discovered that some AMPs manifest antagonistic interactions with hLys, in contrast to prior reports [231]. These results, according to the authors, must be considered for the future development of lysozyme and AMP combination therapies.

As discussed in the previous section considering crop protection, the combination of the synthetic undecapeptide BP100 with lysozyme is another example of the synergistic application of lysozyme for the treatment of fire blight [22,226].

The presence of a peptide, inhibiting the growth of *Salmonella enterica*, *Aeromonas salmonicida*, and *Vibrio anguillarum*, was identified in salmon mucus and blood. This peptide correlates with the increase in lysozyme activity following a disease challenge. Two synthetic peptides, homologous of the identified peptides, were prepared and their antimicrobial assayed. The two artificial peptides were devoid of activity against *V. anguillarum* and *A. salmonicida*, but were able to potentiate lysozyme, proving support to the synergy between lysozyme and the identified peptide [232].

Bacteriocin AS-48 is an AMP produced by *E. faecalis* and is active against several Gram-positive bacteria. This AMP is a ribosomal synthesized and post-translationally modified peptide (70 amino acid residues) which undergoes head-to-tail cyclization, affording a cyclic molecule. In a 2018 article [233], its activity against *Mycobacterium tuberculosis*, the agent that causes tuberculosis, was demonstrated. The activity of AS-48 alone and in combination with lysozyme or with ethambutol, a first-line drug used in the treatment of tuberculosis, was studied. The test showed that lysozyme strongly synergized with AS-48 in in vitro cultures, whereas no synergistic effect was observed in the intracellular model of infections. The authors suggested that either lysozyme could not enter the macrophages and reach the intracellular bacteria, or that the enzyme might have been degraded by the macrophage. A strong synergy was found in both systems with ethambutol.

### 4.2. Lactoferrin

Human lactoferrin is a mucosal glycoprotein present in milk and in secretions, such as saliva, tears, serum, and neutrophiles granules. It is endowed with bacteriostatic and antimicrobial activities. The bacteriostatic effect is due to its ability to sequester iron, an essential ion for bacterial growth. The iron-free molecules of lactoferrin (ALF), when they undergo proteolysis, produce cationic peptides (lactoferricins) that can destabilize microbial membranes. The alteration of the permeability of the external membrane due to lactoferrin can facilitate the action of lysozyme on the peptidoglycan [234].

This already known synergistic action was investigated in 2015 [235] against *Streptococcus pneumoniae*, a colonizer of the nasopharynx of healthy children and adults, from where it can spread to lungs, meninges, and blood, leading to pneumonia, meningitis, or septicemia. Since lysozyme is a protein present at the mucosal sites, the potential synergistic effect with ALF was examined. The results showed that the combined effect of ALF and lysozyme was significantly higher than that of each protein, alone, for one of pneumococci strains examined. In other cases, the combination was not statistically more effective. Synergistic or additive killing by antimicrobial agents, including lactoferrin present in the human airway surface liquid, was investigated in 2000 [236]. The authors examined the combination of lysozyme with lactoferrin and the secretory leucocyte protease inhibitor (SLP1). The triple combination of lysozyme, lactoferrin, and SLP1 showed a greater synergy.

### 4.3. EDTA

Ethylenediaminetetraacetate (EDTA) has been used in several food products as a chelating agent to prevent oxidation. Chelating property to divalent cations like Ca^2+^ and Mg^2+^ is responsible for its antibacterial activity. Indeed, EDTA inhibits the growth of microorganisms by depriving Mg^2+^, Ca^2+^, and Fe^2+^, which are essential for microbial growth. Furthermore, chelating agents contribute to the detachment of cells from the biofilm and to the killing of biofilm-producing microorganisms by depriving Mg^2+^ associated with lipopolysaccharides (LPS).

The works of Boland et al. [237,238] demonstrated that the addition of EDTA enhanced inhibitory effect of lysozymes on strains of *E. coli*. The level of inhibition achieved with the addition of EDTA was 10 times as high as that achieved, in an equal concentration, with disodium pyrophosphate (DSPP) and pentasodium tripolyphosphate (PSTPP). DSPP and PSTPP were chosen as potential EDTA replacement chelators since they are negatively charged polyanions that form complexes with cations, such as copper, calcium, iron, zinc, and magnesium. Even if each lysozyme–chelator combination was effective, the concentrations of phosphate required to increase the lysozyme activity against *E. coli* were higher than the EDTA concentrations showing the same effect. The same authors [237] also studied the effect of different concentrations of mono-, di-, and trivalent cations on the antimicrobial effect of lysozyme–EDTA combinations. Calcium and magnesium resulted in the production of important ions for the reversal of lysozyme–chelator activity against *E. coli* strains, probably due to their role in the stability and structure of the LPS layer. Iron was effective in neutralizing lysozyme–chelator inhibition, probably because of its manifold functions and binding constants to chelators. Sodium and potassium showed a variable- and strain-dependent behavior. The study demonstrated that the efficacy of the lysozyme–chelator combination depended on the composition of the food to be protected.

According to a 2004 study [239], the addition of EDTA enhanced lysozyme activity against *Listeria monocytogenes*, while lysozyme alone inhibited the growth of *L. monocytogenes* Scott A, but did not inhibit growth of other *L. monocytogenes* strains. In the same work, the enhancement of lysozyme activity against *E. coli* by the addition of EDTA was also described, and the results were in agreement with the results of Boland et al. [237,238].

### 4.4. Antibiotics

The rapidly growing resistance of pathogenic bacteria to conventional antibiotics prompted the continuous research of new drugs or combinations of conventional antibiotics with new candidates. Antimicrobial peptides and proteins of the innate immune system constitute a possible approach.

In this context, an Indian team studied the combination effect of nine antibiotics with lysozyme and serratiopeptidase against six different bacterial cultures. While the results for the combination with serratiopeptidase were encouraging, the combination of lysozyme and fluoroquinolone gatifloxacin (the more active antibiotic when compared to the other antibiotics in all the cultures) was ineffective [240]. More recently, other fluoroquinolones, in combination with lysozyme, were the subject of DFT calculation and docking, to study their interaction with lysozyme. In 2018, ciprofloxacin and levofloxacin were investigated in antimicrobial assays and an antagonistic effect of lysozyme in an acid medium was evidenced [241].

In 2017, the location of different prototrophic forms of norfloxacin in lysozyme were examined with molecular docking simulations [242]. An analogous study was recently carried out to investigate the interaction between lysozyme and cephalosporin ceftazidime, by means of DFT calculations and docking [243]. The article did not report any antimicrobial activity data.

The antimicrobial activity of the combination of rhLys and tobramycin against *Pseudomonas aeruginosa* was assayed in a hamster model, in vitro [244]. Tobramycin is a potentially hazardous antibiotic, usually delivered by nebulization. The authors planned to study the combined aerosol delivery of both agents to verify if the addition of rhLys can lower the dose of the drugs and, therefore, reduce the harmful effect and improve patient outcomes. Hamsters instilled with *P. aeruginosa* were treated with an aerosol containing 1% rhLys, 3 μg tobramycin, or both agents. To identify the contribution of rhLys to anti-bactericidal activity, the amount of tobramycin used in the experiments was equivalent to 1% of the standard dosage. The results showed an improvement to all examined parameters in the animals treated with both components, compared to those receiving rhLys or tobramycin alone. *Pseudomonas aeruginosa* incubated in vitro with both rhLys and tobramycin showed less viability than those treated with either agent alone.

*P. aeruginosa*, a cause of nosocomial infections and of chronic respiratory infections in patients with cystic fibrosis, was also the object of a study aimed to improve the modest basal effect of lysozyme (from egg whites) by subinhibitory doses of colistin, an antibiotic of the polymyxin family and active as a permeabilizing agent [245,246]. *P. aeruginosa* has an outstanding capacity for the development of antibiotic resistance through the chromosomal mutations of factors linked to peptidoglycan recycling, which frequently causes resistance to the antipseudomonal β-Lactams. The synergy between lysozyme and colistin, due to their action on the cell wall, is shown in Table 2.

Bactericidal synergism between daptomycin, a lipopeptide antibiotic, and Cpl-1, the bacteriophage Cp-1 lysozyme, against *Streptococcus pneumoniae* was documented both in vitro and in a mouse model [247]. Mice were treated intraperitoneally with an extremely virulent serotype of *S. pneumoniae*. Subtherapeutic doses of daptomycin (0.4 mg/Kg) and Cpl-1 (0.4 mg/Kg and 0.1 mg/Kg) were administered either alone or in combination, 1 h after the bacterial challenge. Daptomycin, in combination with lysozyme, increased the percentage of surviving mice on day 7 (80%) compared with the untreated control (0%), daptomycin or Cpl-1 monotherapy (35% and 0%, respectively).

Metronidazole is an antibiotic that has been used against *Helicobacter pylori*, a human pathogen that plays an important role in peptic ulcers and chronic gastritis. The reduced efficacy of metronidazole, due to the onset of resistance in *H. pylori*, can be overcome by increasing the doses and prolonging the duration of therapy. In order to improve its activity and lower the resistant mutant, a prevention therapy using a combination of metronidazole with hLys was studied [248]. The choice of lysozyme, even if *H. pylori* is resistant to this enzyme, was explained by the authors with its ability to modify the permeability of bacterial cells and to promote the antibiotic adsorption. Its effect on the outer and inner membranes was detected by a fluorescent probe. Lysozyme showed to enhance cell membrane permeability at a concentration of 0.3 mg/mL. The MIC of metronidazole against *H. pylori* was reduced from 8 to 4 μg/mL and the mutant prevention concentration from 153.6 μg/mL to 25.6 μg/mL.

The use of a combination between antimicrobial peptides, proteins, and conventional antibiotics was also the way to combat antibiotic resistance and proposed by a Russian team in 2019 [249]. The egg white lysozyme was studied in combination with glutamicin, ofloxacin, oxocillin, rifampicin, and polymyxin B against selected bacteria. The inhibitory concentrations indexes were evaluated using the checkerboard titrations method. The results are summarized in Table 3 and Table 4.

A combination of linezolid, an antibiotic of oxazolidinones, and lysozyme was loaded and coated onto a metal organic framework (MOF) was suitably functionalized, and the antimicrobial property of the obtained system against *Staphylococcus aureus*, *E. coli*, and methicillin-resistant *S. aureus* was evaluated [250]. The chosen MOF was UiO-66-NH_2_, a metal organic compound made of [Zr_6_O_4_(OH)_4_] clusters with 1,4-benzodicarboxylic acid struts. By means of an amidation reaction, a photosensitizer (zinc phthalocyanine) was bound to the MOF and linezolid. Lysozyme was then loaded in the pores and on the surface of the MOF. The immobilized lysozyme could specifically react with the bacterial cell wall, allowing the nanomaterials to enter the bacteria more easily. The cytotoxic singlet oxygen, generated by the irradiation of the photosensitizer, in combination with immobilized linezolid and lysozyme, resulted in an in vitro potent antibacterial effect.

#### L-Form Bacteria

In this section, the synergy between conventional antibiotics and lysozyme was described. The combination of β-Lactams and immune enzymes, such as lysozyme, causes an unexpected effect, selecting for viable, wall-less bacteria. The peptidoglycans (PG) of the bacterial cell wall were synthesized and their dynamic structure maintained by the action of penicillin-binding proteins (PBPs) and autolysins, which build and hydrolyze PG, respectively. β-Lactams killed the bacteria since it bound and inactivated PBPs, preventing the PG synthesis. Lysozyme, on the contrary, cleaved the glycan structure and degrading PGs. Many bacteria, both Gram-positive and Gram-negative, can convert from rods into a spherical wall-deficient state, the “L-form”, completely resistant to β-Lactams. The presence of L-forms was identified in humans, animals, and plants. 

**Figure 5:**
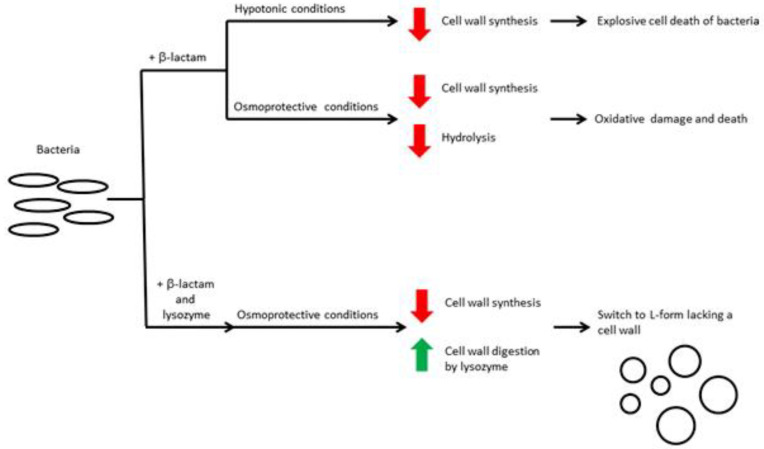
Various fates of bacteria upon β-Lactam treatment.

The L-forms were discovered 80 years ago and, in 2018, Kawai et al. [251] published the results of their work aimed to explain the combined action of β-Lactams and lysozyme. Penicillin alone blocks L-form formation, inhibiting both synthesis, as previously reported in literature, and hydrolysis, which is essential, according to the authors, for inducing L-forms. The bacteria could exploit the host lysozyme to escape antibiotic-mediated killing by switching to an L-form. In a review published in the same year, Burke [252] collected in a scheme the various fates of bacteria upon β-Lactam treatment, depending on their environment and the presence or absence of lysozyme (Figure 5).

### 4.5. Phages

Vibriosis, caused by the Gram-negative bacteria of the genera *Vibrio*, is a common disease in marine and freshwater fish, both in natural and commercial production systems. *Vibrio parahaemolyticus* is an important human pathogenic bacterium. Its presence in the marine environments is frequently the cause of acute gastroenteritis in humans, after the consumption of raw or undercooked seafood. To avoid the administration of antibiotics in aquaculture, phage therapy is an interesting alternative and is well documented in literature. Phage genome injections into the host cell is facilitated by the lytic enzymes that digest the cell wall from the outside. Starting from these considerations, the effect of the external addition of a lytic enzyme, such as lysozyme from chicken egg whites to the phage, was studied [253]. Three phages (VP-1, VP-2, and VP-3) were isolated using *V. parahaemolyticus* as a host. The assays were performed with single-phage suspension and with a combination of the phage with lysozyme. The results showed that the addition of lysozyme to the samples had a positive effect on the phages’ activity. The extent of the activity increase was dependent on the type of phage and on the lysozyme concentration. The three phages, in the presence of lysozyme (10 mg/mL), showed a higher efficiency to inactivate *V. parahaemolyticus*, in comparison to the results obtained with the phages alone. The lytic effect was not observed if lysozyme was used alone, but lysozyme improved the phage entrance in the host cells. VP-3 is the more effective phage and the addition of lysozyme showed only a small improvement, when compared with the other less active phages, VP-1 and VP-2. The bacterial inactivation of these two phages was seven and two times faster, respectively, in the presence of lysozyme, when compared with the results observed with the phages alone. The positive effect observed by adding lysozyme was explained with the increase in the number of phages produced by the bacteria. The same effect caused by the addition of 10 mg/mL of lysozyme to VP-1 and VP-2 was observed by the addition of 3 mg/mL to VP-3.

### 4.6. Selenium Nanoparticles 

Selenium is an essential trace element in the diet of living creatures at low concentrations, but it becomes toxic at higher concentrations. The least toxic form of selenium is elemental Se. Se nanoparticles are active against pathogenic bacteria, fungi, and yeast.

In a 2020 study [254], the interaction between selenium nanoparticles (SeNP) and lysozyme (from chicken egg whites) was investigated to verify if the nanohybrid (SeNP + Lys), with the nano and bio counterparts, can be endowed with a synergistic effect. The nanohybrid was analyzed using the dynamic light scattering technique to establish the sizes of the nanoparticles and circular dichroism spectropolarimetry to verify that lysozyme retained its native conformation, which is strictly related to the catalytic activity. The antibacterial efficiency of the individual components of the nanohybrid was tested against *S. aureus* and *E. coli*. The MIC of SeNPs against *S. aureus* was 82 μg/mL. The MIC of lysozyme was 10 mg/mL. Neither the nanoparticles nor the enzyme completely inhibited the growth of *E. coli*: the highest inhibition (41%) was observed at 660 μg/mL of SeNPs and 2 mg/mL of lysozyme. The nanohybrid containing 50 and 100 μg/mL of lysozyme and the lowest amount of SeNPs induced the complete inhibition of *S. aureus*. Three low concentrations of SeNPs (1, 5, 10 μg/mL) with lysozyme were able to significantly increase the antibacterial activity against *S. aureus*, with respect to the protein alone. In the case of *E. coli*, the nanohybrid system with 330 μg/mL SeNPs and 2 mg/mL lysozyme presented the highest inhibition level (74%). It was observed that both the nano and bio components acted synergistically in the nanohybrid.

### 4.7. Plant Flavones

**Figure 6:**
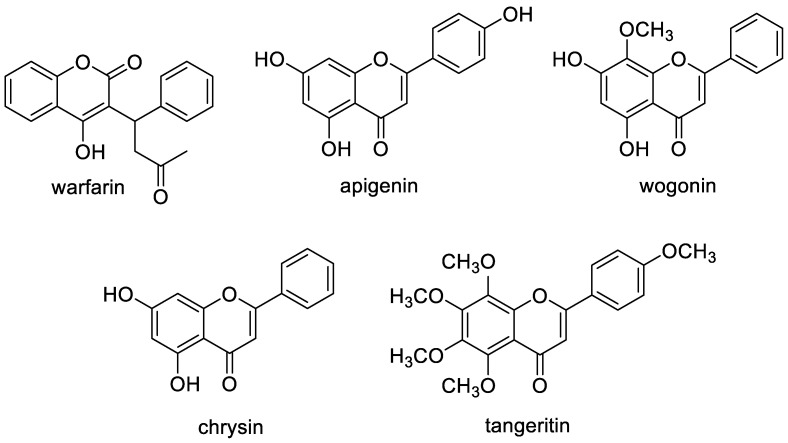
Chemical structure of some flavones.

Flavones are a class of plant secondary metabolites endowed with anti-inflammatory and antibacterial effects (Figure 6).

The flavone apigenin, isolated in bee propolis and chamomile flowers, inhibits the enzyme D-ala-D-alanine ligase, which catalyzes the formation of the dipeptide D-ala-D-ala, the precursor of the peptidoglycan. Apigenin exerts a synergic action with β-Lactam against methicillin-resistant *S. aureus*. Similar antimicrobial effects have also been shown by other flavones, such as chrysin, warfarin, and tangeritin. The antimicrobial effect of flavones on three nasal pathogens frequently found in chronic rhinosinusitis patients, the yeast *Candida albicans*, the Gram-positive *Staphylococcus aureus*, and Gram-negative *Pseudomonas aeruginosa* was investigated in 2017 [255]. The very subtle effects observed in the case of *C. albicans* and *S. aureus* were in contrast with the more marked effect in *P. aeruginosa*. A flavones mixture significantly reduced planktonic growth and the culture optical density at 600 nm (OD_600_), a measure of bacteria lysis. The effect of flavones on the efficacy of airway antimicrobials, such as lysozyme, against *P. aeruginosa* was also tested in the same study. The rapid lysis of *P. aeruginosa* (decrease in OD _600_) was detected in the presence of rhLys and flavones. A mixture of flavones (100 μM each) significantly enhanced lysozyme-mediated *P. aeruginosa* lysis, while individual flavones did not enhance lysozyme-mediated lysis.

### 4.8. Galleria Mellonella Apolipophorin III

Apolipophorin III is a hemolymph protein functioning in the lipid transport and immune response in insects. It is active against Gram-positive and Gram-negative bacteria. In a 2013 article [256], the synergistic action of Apolipophorin III from *Galleria mellonella* with lysozyme was investigated. The authors demonstrated that the permeabilizing activity of lysozyme toward *E. coli* cells increased significantly in the presence of Apolipophorin III, which was devoid of the ability to perforate the bacteria when used alone. 

## 5. Lysozyme Modifications

The wide application field of lysozyme, with its antibacterial specificity against certain Gram-positive bacteria and, to a less extent, against Gram-negative bacteria, prompted the study of lysozyme modifications. The research of modified lysozyme forms is finalized to achieve more active or more stable lysozyme derivatives. The modifications can affect the chemical structure of the protein or its physical properties, mainly electric charges.

### 5.1. Immobilization 

Immobilization is a traditional method aimed at obtaining more stable and easier to handle enzymes [257]. The immobilization techniques used to or within support can be classified as physical (weak interactions between the support and the enzyme) or chemical (covalent bonds formation between the support and the enzyme). The physical methods include the adsorption on the surface (hydrophobic, electrostatic interactions, and hydrogens bonds) and the entrapment in a structure chemically inert toward the protein. The chemical methods can exploit functional groups of the protein to form covalent bonds with the support, or bifunctional reagents, such as glutaraldehyde, which act as a link between the enzyme and the support. The use of bifunctional reagents can also lead to the formation of the so-called cross-linked enzyme aggregates (CLEAs) [258]. 

The obtained lysozyme derivatives showed a decreased antibiotic activity against *M. luteus* in respect to the native lysozyme, but, at times, the modified lysozyme also became active against some Gram-negative microorganisms.

In Table 5, several immobilized lysozyme preparations active against several reported microorganisms are collected. For each example, the immobilization technique and the possible applications are briefly described.

Lysozyme was also immobilized on various nanostructured materials by adsorption or covalent attachment [291]; in Table 6, several already developed applications or future promising uses are reported.

### 5.2. Chemical Modifications

The peptide chain of lysozyme can be modified by a proteolytic transformation aimed to isolate fragments still enzymatically active or showing a different activity, probably due to the electric charges present on the protein surface, against Gram-negative bacteria and/or other microorganisms [328,329]. An opposite approach provides the elongation of the chain by the fusion with different material or sulfidic proteins or peptides. Moreover, conjugates or complexes of lysozyme derivatives can be obtained by a reaction with polysaccharides and fatty acids with the aim to modify the hydrophobicity or the electric charges of the protein. Finally, the modifications of functional groups present in the side chain of some amino acid residues by oxidation or reduction are reported in literature.

Several examples of chemical modifications, improving the lysozyme activity, and the related potential applications are reported in Table 7.

### 5.3. Physical Modifications 

The thermal treatment of lysozyme is the most commonly reported physical process. By heating a lysozyme sample, the formation of oligomers, mainly the dimer, is, often, observed to be more active than the monomer. By subjecting the bacteria to high hydrostatic pressure, an improved sensitization to lysozyme can be achieved. The development of variants showing a modified charge on the protein chain, pave the way to an improved activity against Gram-negative bacteria, such as *P. aeruginosa*, evading the electrostatic inhibition by anionic biopolymers, associated with airway inflammation. The most interesting results about physical modifications are summarized in Table 8.

## 6. Conclusions

The impressive number of literature data, published in the last two decades, dealing with lysozyme antimicrobial activity clearly proves the great interest provoked by this well-known molecule. Lysozyme is often regarded as a potential help to overcome the problem of traditional antibiotic resistant bacterial infections. This interest explains the extensive research of lysozyme modifications to improve the applications in medicine, veterinary, crop production, feed, and food preservation.

Many works are focused on lysozyme engineering to penetrate the outer membrane, the main obstacle to its activity against Gram-negative bacteria. Modifications of the dense positive charge of hLys, defined as “Achilles’ heel” in a 2011 article [377], can aid the development of novel therapeutic applications. Lysozyme containing coatings and thin films, obtained through nanotechnology approaches, can provide the response to the problem of food-borne pathogens or biofilm formation on medical devices. The availability of transgenic rice, expressing hLys, is a promising supplement source for infant formula, in breastfeeding difficulty or impossibility.

In conclusion, it appears appropriate to conclude this review with Alexander Fleming’s famous quote: “We shall hear more about lysozyme” [378].

## Figures and Tables

**Table 1:** Relevant data on the synergic uses of different substances with lysozyme, in order to enhance the susceptibility of Gram-negative bacteria.

Combined Use with	Application/Claimed Use	Antibiotic Activity Evaluated Against/On	Refs.
Cinnamaldehyde	Storage of olive flounder (*Paralichthys olivaceus*) fillets/lowered total viable count	*S. putrefaciens* and *P. fluorescens*	[177]
Acidic electrolyzed oxidizing water	To prolong the shelf life of carp fillets from a microbiological point of view	Total viable count, *Enterobacteriaceae* count, and anaerobic mesophilic count	[178]
Heat treatment and dilution with hydrogen peroxide associated with packaging in a controlled atmosphere	To extend the shelf life of pork meat by more than 20%, compared with the control sample without lysozyme	Aerobic plate count, *Enterobacteriaceae*, *Pseudomonas* spp., and lactic acid bacteria	[179]
High hydrostatic pressure	Not significant enough to differentiate lethality of lysozyme without additives in cheeses made from raw milk	*B. cereus*	[180]
Disodium ethylenediaminetetraacetate salt	Buffalo meat in refrigerated conditions	Total viable mesophilic count, total viable psychrotrophic count, lactic acid bacteria, *Pseudomonas* spp., and *B. thermosphacta*	[181]
Chitooligosaccharides	The microbiological quality of minced meat stored in refrigerated conditions was improved	*Escherichia coli*, *Pseudomonas fluorescens*, *Bacillus cereus*, and *Staphylococcus aureus*	[183]
Ethylenediaminetetraacetic acid disodium salt	To reduce the growth of *Y. enterocolitica* in orange beverages	*Y. enterocolitica*	[188]
Layer-by-layer chitosan-organic rectorite composites and negative charged sodium alginate.	The sensory analysis and physicochemical analysis applied to assess the effects of layer-by-layer film coating confirmed a higher score for the packaged pork (4 °C for 21 days)	*E. coli* and *S. aureus*	[184]
EDTA in anti-microbial starch-based active food packaging films	Cooked rice with pulses/effective to inhibit the growth of spoilage microorganisms	Contaminated from the open environmental sources (mainly Gram-negative cocci)	[185]
EDTA	A different lysozyme activity against the tested microorganisms was observed, increasing the ratio of lysozyme/EDTA from 11.14:8.14 mg/mL to 11.14:14.14 mg/mL.	*Micrococcus lysodeikticus* and *Escherichia coli*	[182]
Enterocin AS-48	Synergy confirmed in liquid whole eggs, egg whites, and egg yolks, at 4 °C and 28 °C	*B. cereus* and *S. aureus*	[186]
Pomegranate peel extract	To maintain the quality of mackerel fillets wrapped with gelatin/polycaprolactone composite film and to prolong the shelf life of the product	Total mesophilic counts and psychrotrophic bacteria counts	[187]

**Table 2:** Susceptibility to colistin and lysozyme, and the combined treatment (lysozyme + colistin) of two strains of *Pseudomonas aeruginosa* (PAO1 and PA14).

Strain	Colistin MIC (μg/mL)	Percentage of Bacterial Survival after Each Treatment ^a^		
		Colistin (0.1 μg/mL)	Lysozyme (25 μg/mL)	Lysozyme + Colistin (25 + 0.1 μg/mL)
PAO1	0.75	45.3 ± 11.2	35.9 ± 8.0	4.1 ± 1.3
PA14	0.25	19.1 ± 3.9	52.3 ± 10.2	3.0 ± 0.62

^a^ Percentage of survival after each treatment in respect to the initial inoculum. PAO1 and PA14 survival percentages in the lysozyme assay buffer alone were between 80–90% after incubation.

**Table 3:** Antimicrobial activity of individual fractions of lysozyme and antibiotics against Gram-negative and Gram-positive bacteria.

	MIC ^a^ (μM) toward Bacterial Strains			
	Gram-Negative		Gram-Positive	
Sample	*E. coli* ML-35po	*A. baumannii*	MRSA ATCC 33591	*M. luteus* CIP A270
Lysozyme	>250 (500)	1.2	>250 (500)	0.08
Oxacillin	>250 (500)	25	7.5	3.1
Polymyxin B	0.4	1.25	12.5	0.6
Gentamicin	0.625	0.06	0.16	0.16
Rifampicin	10	0.125	0.003	0.003
Ofloxacin	0.125	0.3	0.625	5

^a^ MIC values are medians of 3–6 independent experiments made in triplicates. If the actual MIC value was out of the tested concentrations range, it was assessed as twice the maximal tested concentration; the corresponding value is given in square brackets.

**Table 4:** Antimicrobial activity of combinations of lysozyme with antibiotics against Gram-negative and Gram-positive bacteria.

	Minimal FICIs ^a^ of the Lysozyme\Antibiotic Combination (AB) against Bacteria										
	Gram-Negative					Gram-Positive					
AB	RIF	PMB	GEN	OFL	OX	AB	RIF	PMB	GEN	OFL	OX
*E. coli* ML-35p						MRSA ATCC 33591					
Lysozyme	0.62	0.25	0.53	1.12	-	Lysozyme	0.62	0.56	0.53	1.12	0.53
*A. baumannii*						*M. luteus* CIP A270					
Lysozyme	1	0.5	0.75	1	1	Lysozyme	0.75	0.62	0.62	0.62	0.75

RIF, rifampicin; PMB, polymyxin B; GEN, gentamicin; OFL, ofloxacin; and OX, oxacillin. ^a^ Fractional inhibitory concentration indices (FICI) = [A]/[MIC A] + [B]/[MIC B], where [A] and [B] are the respective concentrations of substances A and B in their combination, effectively inhibiting bacterial growth; FICI > 2 indicates antagonism, 1 < FICI ≤ 2 shows independent action, 0.5 < FICI ≤ 1 corresponds to additivity, and FICI ≤ 0.5 denotes synergy.

**Table 5:** Immobilization of lysozyme.

Support	Tested Microorganisms	Reagent or Immobilization Technique	Applications/Claimed Uses	Refs.
Vitreous surface	*E. coli*		Water decontamination	[259]
Wool fabric	*S. aureus*	Glutaraldehyde	Antibacterial functionalization for textile goods	[260]
Wool fabric	*E. coli*	Tris(hydroxymethyl)phosphine	Antibacterial functionalization for textile goods	[261]
Cotton fabric	*M. lysodeikticus*	Glycine esterified	Medical hygiene	[262]
Silk textiles	*S. aureus* *E. coli*	Physically adsorbed	Functional wound dressing	[263]
Polyacrylonitrile membranes	*S. aureus*	Glutaraldehyde	Water treatment and food manufacturing	[264]
Polyacrylonitrile membrane hydrolyzed	*E. coli* *S. aureus*	Layer-by-layer self-assembly	Antibacterial thin film composite membranes for aqueous molecular separation	[265]
Polystyrene/poly (styrene-10-maleic anhydride)	*S. aureus*	CLEA	Antimicrobial process in biomedical and engineering industries	[266]
Polyethylene glycol	*L. ivanovii* *M. luteus*	Reductive amination on pretreated stainless steel surface	Antifouling agents	[125]
Poly(3,4-ethylenedioxythiophene) (PEDOT)	*S. epidermidis*	Incorporations in films	Regeneration of tissues	[267]
Cellulose acetate	*S. aureus*	Electrostatic adsorption, electrospraying	Food packaging and antimicrobial wound dressing	[268]
Gelatin/sodium carboxymethylcellulose	*S. aureus* *P. aeruginosa* *E. coli*	Inclusion in a polymeric matrix	Mucoadhesive form of lysozyme	[269]
Alginate, iron cations	*M. luteus*	Entrapment	Food packaging materials	[270]
Chitosan	*S. aureus* *B. subtilis* *S. flexneri* *P. aeruginosa*	CLEA	Repeated uses as antimicrobial material	[271]
Chitosan/alginate	*E. coli* *S. aureus*	Hydrogel	Food industry	[272]
Calcium phosphate on chitosan	*M. lysodeikticus*	Incorporation	Bone tissue engineering	[273]
Xanthan	*M. luteus*	Hydrogel	Wound dressing	[274]
Agarose, free amino acids	Gram-positive Gram-negative	Reductive amination	Blood plasma and whole blood purification by extracorporeal therapy procedures	[275]
Agarose, sericin	*E. coli* *S. aureus*	Gel	Wound dressing	[276]
Polysaccharides particles or liposome	*B. subtilis* *M. luteus* *E. coli* *S. marcescens*	Encapsulation with herbal extracts	Food preservation and wound healing	[277]
Exopolysaccharides	*E. coli*	Incorporation into films	Biodegradable coatings for fruits and vegetables	[278]
Graphene oxide	*E. coli*	Electrostatic interactions	Antibacterial membranes	[279]
Graphene oxide, polydopamine	*E. coli*	Electrostatic and hydrogen bond interactions	Medical treatments and food safety fields	[280]
Capsid	*M. luteus*	Encapsulation	Nanoreactor at physiological conditions	[281]
Balsa	*E. coli* *S. aureus*	Encapsulation	Wound healing	[282,283]
Calcium carbonate	*M. lysodeikticus*	Encapsulation	Catalysis, disease treatment, and tissue engineering	[284]
Layered double hydroxide	*E. coli* *S. aureus*	Van der Waals forces	Wound healing	[285]
Silica	*E. coli* *S. aureus*	Entrapment	Coating containers used to store chirurgical devices, catheters, implants, artificial prosthetics, and other materials to reduce hospital infections	[286]
Laser sintered titanium	*S. gordonii* *S. sanguis*	Layer-by-layer self-assembly	Biofilm inhibition	[132]
Niclosamide	MRSA MERS-COV SARS-CoV-2	Embedded in h-Lys for inhalation route	Delivery to the upper and lower respiratory tracts	[287]
Lactic acid	*B. cereus* *E. coli* *S. typhimurium*	Gelled egg white powder	Food industry	[288]
Microbubbles, immobilized gold NPs	*M. lysodeikticus*	Pressurized gyration	Biosensor for the detection of analytes in aqueous solutions	[289]
Microbubbles, immobilized gold NPs, and polyvinyl alcohol	*E. coli*	Pressurized gyration	Diagnostic tools and environmental bioassays	[290]

**Table 6:** Lysozyme modifications and nanotechnologies.

NANOMATERIAL				
	Tested Microorganisms	Reagent or Method	Applications/Claimed Uses	Refs.
Cellulose nanocrystals	*M. lysodeikticus* *Corynebacterium* sp. *E. coli* *P. mendocina*	Glutaraldehyde	Improved antibacterial action also against Gram-negative bacteria	[292]
Cellulose nanocrystals	*B. subtilis*	Cellulose-CHO	Inhibition of biofilm	[110]
Cellulose acetate nanofibers, sodium alginate	*S. aureus*	Electrostatic interactions	Milk and dairy products	[293]
Cellulose acetate nanofibrous LBL	*E. coli* *S. aureus*	Electrostatic interactions	Food packaging, adhesive wound dressing, and tissue engineering	[294]
Cellulose NPs/b-chitosan	*L. innocua* *E. coli*	Encapsulation	Packaging material for shelf life extension	[295]
Nanocellulose aerogel	*S. aureus* *E. coli*	Electrostatic interactions	Wound dressings	[296]
Chitin nanowhiskers	*E. coli* *S. aureus* *B. subtilis*	Adsorption	Enhancement of the antibacterial efficiency for food preservation	[297]
Chitosan NPs/tannin	*S. aureus* *S. enteriditis* *L. monocytogenes*	Encapsulation	Food industry	[298]
Chitosan NPs	*S. epidermidis*		Encapsulation of antimicrobial peptides	[299]
Poly-γ-glutamic acid, chitosan NPs	*E. coli* *B. subtilis*	Loading	Controlled delivery system	[300]
Polystyrene nanospheres	*E. coli*	Electrospinning	Food processing and medical equipment	[301]
Nanopatterned poly(isopropylacrylamide)	*E. coli* *S. epidermidis*	Adsorption	Mitigation of short-term bacterial biofouling	[302]
Eugenol-casein NPs	*S. aureus* *Bacillus* sp.	Encapsulation	Food preservation	[303]
Melanosome nanostructures	MRSA	Electrostatic interactions	Tissue repair	[304]
Glass NPs	*B. subtilis*, (human hepatocellular carcinoma)	Electrostatic interactions	Treatment of bone defects caused by tumors	[305]
Carbon NTs single-wallet carbon nanotubes	*M. lysodeikticus*	Noncovalent or covalent interactions	Covalent functionalization led to improved dispersion stability and longer duration of bacterial lysis relative to noncovalent lysozyme single-walled carbon nanotubes	[306,307]
Single walled carbon NTs, DNA fibers	*M. lysodeikticus*	Spinning, electrostatic interactions	Drug delivery, tissue engineering, and biocompatible composites	[308]
Nanodiamonds	*E. coli*	Electrostatic interactions	Biolabel to observe the interaction of Lys with bacteria	[309]
Silica NPs	*E. coli*	Electrostatic interactions	Highly efficient antibacterial agent in vitro and in vivo with low cytotoxicity and negligible hemolytic activity	[310]
Silicon nanowires, poly (methacrylic acid)	*E. coli*	Adsorption	Engineering of surfaces with switchable functionalities	[311]
Halloysite NTs	*E. coli*	1,6-hexan-ethylene diisocyanate	Reduced fouling in water treatment	[312]
Montmorillonite K10-silver NPs	*E. coli* *P. aeruginosa* *MRSA* *L. monocytogenes*	Complexes	Activity against antibiotic resistant bacterial strains	[313]
Montmorillonite-silver NPs	*E. coli* *S. aureus*	Complexes	Production of sorbents with antibiotic properties	[314]
Rectorite nanofibrous membrane	*S. aureus* *E. coli*	Electrospinning	Food engineering and biomedical materials	[315]
Molybdenum disulfide nanosheets	Ampicillin-resistant *E. coli* *B. subtilis*	Electrostatic interactions	Design and synthesis of novel nanozyme antibacterial agents	[316]
Molybdenum disulfide nanosheets	*E. coli* *S. aureus*	Coating	Microfiltration membrane for water purification	[317]
Zinc oxide NPs	*E. coli* *S. aureus*	Glutaraldehyde + aminated ZnO NPs	Biomedical	[318]
Titania nanosheets	*M. lysodeikticus*	Layer-by-layer technique, electrostatic interactions	Antibacterial coatings	[319]
Layered double hydroxide nanocomposites	*E. coli* *B. subtilis*	Loading	Water purification processes	[320]
Gold capped nanoclusters with ampicillin	MRSA		Wound healing	[321]
Gold NPs	*B. subtilis*	Tryptophan residues of Lys + N-bromo succinimide	Drug delivery and bioimaging	[322]
Silver NPs		Tyrosine residues of Lys + N-acetyl imidazole		
Gold NPs	*S. epidermidis* *E. coli*	Layer by layer	Long-term antibacterial coating, biocatalysis, and biosensor	[323]
Gold NPs	*Acinetobacter baumanii* *Enterococcus faecalis*		Bacterial labeling and antimicrobial agents against antibiotic resistant bacteria	[324]
Gold NPs	*S. aureus* *E. coli*	Hybrid film	Killing and removal of adherent bacteria on the surfaces of medical devices	[325]
Silver NPs	*E. coli* *S. aureus* *B. anthracis* *C. albicans* resistant *P. mirabilis*	Nanoparticles from stable colloid (silver acetate and lys in methanol)	Aseptic and therapeutic use	[326]
Silver NPs in nanogel (dextran + lys)	*E. coli* *S. aureus*	Maillard reaction (nanogel), AgNPs embedded in nanogel	Inhibition of biofilm formation	[327]

**Table 7:** Chemical modifications of lysozyme.

**FRAGMENTATION**				
	**Microorganisms**	**Method**	**Applications/Claimed Uses**	**Refs.**
Nine amino acids	Viral infection	Clostripain	HIV infection and inhibition of tumor growth	[330]
Bactericidal domain from human milk Lys	*E. coli* *S. aureus* *C. albicans*	Pepsin, pH 4	Treatment of microbial infections	[20]
From c-type Lys	Gram-positive Gram-negative	Pepsin, pH 4	Treatment of microbial infections	[331]
From goose egg white Lys	*E. coli* *B. bronchiseptica* *S. enteridis* *H. pylori* *S. aureus* *S. epidermidis* *B. subtilis* *M. luteus*	Pepsin, pH 2	Treatment of microbial infections	[16]
From hen egg white Lys	*E. coli* *S. carnosus*	Pepsin, pH 2	Treatment of microbial infections	[332]
Pentadecapeptide from chicken egg white Lys	*E. coli*	Clostripain	Treatment of microbial infections and nontoxic against erythrocytes	[333]
**FUSION PEPTIDES and PROTEINS**				
**Fusion Product**	**Microorganisms**		**Applications/Claimed Uses**	**Refs.**
Human β-defensin-3-lysozyme	MRSA *S. aureus*		Therapy of MRSA infection	[334]
Pesticin-N-terminus of T4 lysozyme	*E. coli* *Yersinia*		Therapeutic application to a wide variety of *Yersiniae* and pathogenic *E. coli* strains	[335]
Propeptide of surfactant protein B	*Streptococci* *P. aeruginosa*		Prophylaxis or therapeutic treatment of respiratory and gastrointestinal bacterial infection	[152]
Chimeric polypeptides	*P. aeruginosa*, *K. pneumoniae* *E. coli* *A. baumanii* *S. typhimurium* *S. infantis* *Shigella* *P. mirabilis* *B. thailandensis*		Preparation of new enzymes active against Gram-negative bacteria	[336]
Chimeric phage lysin	*S. pneumoniae*		Treatment of multiresistant pneumococcal infections	[128]
N-terminal hexapeptide-*Sus scrofa* lysozyme	*B. licheniformis* *B. subtilis* *M. lysodeikticus* *S. aureus* *E. coli* *K. pneumoniae* *P. aeruginosa* *S. enteritidis*		Antibacterial lysozyme derivatives as components of food additives.	[337]
**CONJUGATES and COMPLEXES**				
**Starting Material**	**Microorganism**	**Method or Reagent**	**Applications/Claimed Uses**	**Refs.**
Chitosan	*P.aeruginosa* *A. baumannii* MRSA	Maillard reaction	Control of refractory infections	[338]
Chitosan film	*S. faecalis* *E. coli*		Surface coating on perishable fruits and vegetables to enhance microbial safety and extend shelf life of the products	[171]
Chitosan-silicon	*E. coli*		New recyclable antibacterial materials	[339]
Chitosan hydrogel	*S. aureus* *E. coli*	Methacrylate	Tissue engineering and wound healing	[340]
Chitosan, alginate complexes	*E. coli*		Medical preparations characterized by a sustained release and resistance to aggressive environmental conditions	[341]
Dextran sulfate	*S. aureus* *E. coli*	Maillard reaction	Ingredient in formulated food systems or as therapeutic agent	[342]
Xanthan	*M. lysodeikticus* *S. aureus* *E. coli*	Maillard reaction	Functional ingredient with high quality emulsifier, foam producer, or natural antibacterial agent in food	[343]
Ulvan	*S. aureus*	Polyelectrolyte complexes	Nanocarrier for positively charged bioactive molecules	[344]
Oleyl chloride	*E. coli*	Covalent bond (amide)	Increase in hydrophobicity	[345]
Oleic acid	*S. pneumoniae*	Complex	Bactericidal activity against bacterial species with a respiratory tropism	[346]
Transferrin (human)	Gram-positive	Homofunctional linker	Delivery of the composition in the CNS by crossing the blood–brain-barrier	[347]
Monomethoxy polyethylene glycol	*E. coli* *P. aeruginosa*	Succinyl ester of mPEG	Increase in hydrophobicity	[348]
Monomethoxy polyethylene glycol	*M. luteus*	Tetrafluorophenyl 4-(mPEG)-4-oxobutanoate	Sustained release of the hydrophilic lysozyme by passive diffusion	[349]
N-methacryloyl-(L)-histidine methylester	*S. aureus* *E. coli*	Complex	Antibacterial coatings and tissue engineering studies	[350]
Avarone	Gram-positive Gram-negative	Addition to the quinone moiety	Targeting the cell wall vehicle for delivering the quinone	[351]
Cisplatin analogues	*C. albicans* *Cryptococcus neoformans*	Complexes	Antifungal activity	[352]
Pyridylbenzimidazole Au (III)	*C. albicans* *Cryptococcus neoformans*	Complexes	Antifungal activity	[353]
Caffeine, dioctyl sulfosuccinate	*E. coli* *P. aeruginosa* *B. thuringensis*	Colloidal complex	Development of antimicrobial colloidal systems	[354]
Triclosan	*E. coli* *P. aeruginosa* *K. pneumoniae* *S. typhimurium*	Complex	Delivery of phenolic drugs to microbial cells in food and drug systems	[355,356]
Polyproline	*E. coli* *P. aeruginosa*	Recombinant DNA technique	Increase in hydrophobicity	[357]
Pluronic F-127	*B. subtilis*	Reductive amination	Biological systems, including the coating of biomaterials implant surfaces	[358]
Cinnamic aldehyde	*E. coli*	Covalent modification	Increased antibiotic spectrum	[359]
**REACTIONS of LYSOZYME CHAIN**				
**Reaction**	**Microorganism**	**Reagent**	**Applications/Claimed Uses**	**Refs.**
Reduction	*S. aureus* *E. coli* *S. enteritidis*	Sodium sulfite	Increased hydrophobicity, antibacterial agent in food industry	[360]
Oxidation of tyrosine and tryptophan	*M. lysodeikticus*	Reactive oxygen and nitrogen species	Treatment of cancer and infectious diseases, antibacterial, and disinfecting agent	[361]
Oxidation	*P. fluorescens*	Hydrogen peroxide	Increased hydrophobicity	[362]
Arylation	*S. aureus* *S. epidermidis*	Arylation of tryptophan residues with iodobenzene	Increased hydrophobicity	[363]

**Table 8:** Physical modifications of lysozyme.

Physical Process	Microorganisms	Effect	Refs.
Microwave held followed by oxidation	*M. lysodeikticus*	Dimer and trimer formation	[364]
Microwave	*M. lysodeikticus*	Dimer and trimer formation and changes in surface hydrophobicity	[365]
Fluorescens resonance energy transfer	*E. coli*	Variants formation	[366]
Cationic surfactant (gemini)	*M. luteus*	Micelles formation	[367]
High hydrostatic pressure	Gram-negative: *E. coli*, *P. fluorescens*, *S. entericas, S. sonnei*, and *S. flexneri*	Sensitization of bacteria	[101]
High hydrostatic pressure	*E. coli*	Increased outer membrane permeability	[368]
Atmospheric and high hydrostatic pressure	Gram-positive Gram-negative	Sensitization of bacteria	[369]
Thermal treatment (t 80 °C)	*M. luteus* *E. coli*	Formation of dimer depending on pH and concentration	[370]
Thermochemical treatment (60–70 °C + 10–20% H_2_O_2_)	*M. lysodeikticus* *S. epidermidis*	Formation of dimer	[371]
Thermochemical treatment (denaturation with heat or with dithiothreitol)	*E. coli* *S. carnosus*	Oligomers formation	[372]
Dry heating (80 °C, 7 days)	*E. coli*	Increased insertion capacity and ability to induce lipid packing modifications	[373]
Heating of jenny milk	*B. megaterium* *Clavibacter michiganensis* *Clostridium tyrobutyricum* *Xanthomonas campestris* *E. coli*	Antimicrobial activity like synthetic antibiotics against some Gram-positive and Gram-negative strains	[18]
Bioengineered modifications:			
Net charge inversion of a phage lysozyme	*S. pneumoniae*	Mutation of a Cpl-7	[374]
Charge engineered variant of hLys	*P. aeruginosa*	Redesigned electrostatic potential field	[375,376,377]
